# Sixteen new species of *Mazosia* (*Ascomycota*, *Roccellaceae*) from China revealed by molecular and morphological evidence

**DOI:** 10.3897/imafungus.17.200357

**Published:** 2026-08-12

**Authors:** Shu-Hua Jiang, Zong-Ting Yao, Xian-Dong Xue, Marcela E. S. Cáceres, André Aptroot, Jiang-Chun Wei, Ze-Feng Jia, Robert Lücking

**Affiliations:** 1 State Key Laboratory of Microbial Diversity and Innovative Utilization, Institute of Microbiology, Chinese Academy of Sciences, Beijing 100101, China Botanischer Garten und Botanisches Museum Berlin, Freie Universität Berlin Berlin Germany; 2 College of Life Sciences, Liaocheng University, Liaocheng 252000, China College of Life Sciences, Shandong Normal University Jinan China; 3 College of Life Sciences, Shandong Normal University, Jinan 250014, China Departamento de Biociências, Universidade Federal de Sergipe Itabaiana Brazil; 4 Departamento de Biociências, Universidade Federal de Sergipe, Itabaiana, Sergipe CEP: 49.500-000, Brazil Universidade Federal de Mato Grosso do Sul Campo Grande Brazil; 5 Universidade Federal de Mato Grosso do Sul, Campo Grande, Mato Grosso do Sul, Brazil College of Life Sciences, Liaocheng University Liaocheng China; 6 Botanischer Garten und Botanisches Museum Berlin, Freie Universität Berlin, Königin-Luise-Straße 6–8, 14195 Berlin, Germany State Key Laboratory of Microbial Diversity and Innovative Utilization, Institute of Microbiology, Chinese Academy of Sciences Beijing China

**Keywords:** Diversity, foliicolous lichens, *
Mazosia
*, multi-locus phylogeny, new taxa

## Abstract

*Mazosia* is one of the most commonly encountered foliicolous genera, regularly present with high species richness in closed rainforest understory communities throughout the tropics. This first comprehensive multi-locus (ITS-nuLSU-mtSSU) phylogenetic analysis indicates a high level of previously unrecognized diversity, with a 50% increase in lineages recognized in this genus. On the basis of combined molecular and phenotypic data, sixteen new species of *Mazosia* from China are described: *Mazosia centrica***sp. nov**., *M. dimorphoverrucosa***sp. nov**., *M. gelatinospora***sp. nov**., *M. intermedia***sp. nov**., *M. papillosa***sp. nov**., *M. pruinata***sp. nov**., *M. pseudoaptrootii***sp. nov**., *M. pseudocorticola***sp. nov**., *M. pseudomelanophthalma***sp. nov**., *M. pseudopapillosa***sp. nov**., *M. pseudopilosa***sp. nov**., *M. pseudopruinata***sp. nov**., *M. pseudotenuissima***sp. nov**., *M. sinensis***sp. nov**., *M. straminea***sp. nov**., and *M. verrucosa***sp. nov**. A new, world-wide key to the known species of *Mazosia* is presented.

## Introduction

The lichen-forming fungal genus *Mazosia* A. Massal. (*Roccellaceae*, *Arthoniales*) was formally erected by Massalongo in 1854, with *M. rotula* (Mont.) A. Massal. designated as its type species. This genus constitutes a dominant element of foliicolous lichen assemblages inhabiting lowland tropical forests worldwide (Santesson 1952; Lücking 2008). Species of *Mazosia* predominantly form crustose thalli on the surfaces of intact living leaves, while a smaller proportion colonizes bark and bamboo culms. Diagnostic morphological traits include rounded zeorine apothecia, fissitunicate asci, and transversely septate ascospores ranging from fusiform to acicular in shape (Lücking 2008). Their photobionts belong to either *Trentepohlia* or *Phycopeltis*. Most taxa lack detectable lichen secondary metabolites, yet a small subset of species synthesizes psoromic acid and roccellic acid (Kantvilas 2020; Ertz and Lebreton 2024).

To date, 23 foliicolous species are taxonomically accepted within *Mazosia*, several of which are hypothesized to exhibit pantropical geographic distributions (Lücking 2008; Singh and Pinokiyo 2008; Yao et al. 2021a, 2021b). A limited number of taxa also occurs on bark substrates, predominantly smooth bark (Aptroot et al. 2014; Sakata et al. 2017). Ertz and Lebreton (2024) recently described *M. byssoidea* from Guadeloupe, a species characterized by a loose, byssoid thallus texture—a morphological feature never previously recorded in *Mazosia*—and they also provided an updated identification key for corticolous congeners. Up to seven species have been found co-occurring on single leaves, and three to five species are not rare, often in variable combinations and including widespread taxa, such as *M. dispersa*, *M. melanophthalma*, *M. paupercula*, *M. phyllosema*, *M. pilosa*, *M. praemorsa*, *M. rotula*, *M. tenuissima*, and *M. tumidula*.

Despite the well-documented ecological diversity of the genus, no comprehensive multilocus phylogenetic framework incorporating a broad sampling of *Mazosia* species has been generated to date. This study presents the first genus-scale multilocus phylogenetic reconstruction of *Mazosia* based on three molecular markers (ITS, nuLSU, and mtSSU). By integrating DNA sequence data with morphological phenotypic evidence, we reassess species delimitation and quantify the full diversity of the genus. Through this combined morphological and molecular framework, sixteen new species are formally proposed. This discovery drastically expands the documented phylogenetic diversity of *Mazosia* and reveals a rich, previously unrecognized pool of hidden diversity within tropical foliicolous lichen biotas.

## Material and methods

### Material examined

Three hundred and fifty-eight specimens from China and Brazil were examined in this study. These are preserved in the Fungarium-Lichenum of the Institute of Microbiology, Chinese Academy of Sciences (**HMAS–L**), the Fungarium of the College of Life Sciences, Liaocheng University (**LCUF**), the herbarium of the Botanischer Garten und Botanisches Museum und Botanischer Garten Berlin (**B**), and the herbarium of the Universidade Federal de Sergipe in Itabaiana (**ISE**).

### Phenotypic analyses

For morphological and anatomical studies, a LEICA M125 dissecting microscope (Leica Microsystems, Singapore) and a Zeiss Axioscope2 (Carl Zeiss, Göttingen) compound microscope were used. Photographs were taken with an AxioCam MRc5 connected to a Zeiss Imager A2-M2 microscope (Carl Zeiss, Göttingen) for microscopic features. Thin-layer chromatography (TLC) in solvent systems B and C (Orange et al. 2010; Elix 2014) was employed for the detection of lichen substances.

### Phylogenetic analyses

#### DNA extraction and PCR amplification

A modified CTAB method (Rogers and Bendich 1988) was used for DNA extraction. DNA suspended in ddH_2_O, was amplified by the polymerase chain reaction (PCR). The nuclear ribosomal RNA gene region, including internal transcribed spacers (ITS) 1 and 2 and the 5.8S subunit, was amplified using the primer pair ITS5 and ITS4 (White et al. 1990). A portion of the fungal nuclear ribosomal large subunit nuLSU was amplified using the primers NLF-GGCCCGAGTTGTAATTTGCA (newly designed here), NLR-CATCGCCAGTTCTGCTTACC (newly designed here). Partial mtSSU sequences were amplified using the following primers: mrSSU1 (Zoller et al. 1999) and mrSSU3R (Zoller et al. 1999).

PCR reactions were carried out in 25 μl reaction volume and the components used were 3 μl total DNA, 1 μl each primer (10 μM), 12.5 μl 2× Taq MasterMix (Zhou et al. 2018), 7.5 μl ddH_2_O. Amplification was performed using a Biometra T-Gradient thermal cycler. For the lichenized fungi, cycling parameters of ITS was set to an initial denaturation at 95 °C for 5 min, followed by 35 cycles of denaturation at 94 °C for 30 s, annealing at 54 °C for 30 s, extension at 72 °C for 1 min, and a final extension at 72 °C for 10 min. The PCR conditions of LSU included: initial denaturation at 95 °C for 5 min; 35 cycles of denaturation at 95 °C for 1 min; annealing at 56 °C for 45 s; extension at 72 °C for 2 min; and a final extension at 72 °C for 10 min. The PCR procedure of mtSSU comprised an initial denaturation at 95 °C for 5 min, followed by 35 cycles of denaturation at 95 °C for 1 min, annealing at 50 °C for 45 s, extension at 72 °C for 2 min, and a final extension at 72 °C for 10 min. The PCR products were checked on 0.8% agarose electrophoresis gels stained with ethidium bromide and then sent to the sequencing facilities of BioSune Biotechnology Cooperation, China, for sequencing in both directions. Sources of sequenced specimens and GenBank Accession numbers are listed in Table 1.

**Table 1.** Specimens and sequences for phylogenetic analysis of *Mazosia*.

**Species**	**Collection No**.	**Herbarium No**.	**Country**	**ITS**	**nuLSU**	**mtSSU**	**References**
*Dichosporidium nigrocinctum*	Ertz15378	Ertz15378		**-**	KJ524285	KC820665	Ertz et al. 2015
***Mazosia bambusae***	**Aptroot s.n**.	**B600300041**	**Brasil**	**OR176871**	**OR176539**	**OR176694**	**This study**
***M. bambusae***	**Aptroot s.n**.	**B600300093**	**Brasil**	**OR176872**	**-**	**OR176695**	**This study**
***M. bambusae***	**Aptroot s.n**.	**B600300153**	**Brasil**	**OR176870**	**OR176538**	**OR176693**	**This study**
*M. bambusae*	UPS3c	UPS3c		**-**	KJ851057	KJ851008	Frisch et al. 2014
*M. byssoidea*	Lebreton2311	Lebreton2311		**-**	OR725979	OR725977	Ertz and Lebreton 2024
*M. carnea*	BR15684	BR15684		**-**	KJ524308	**-**	Ertz et al. 2015
*M. carnea*	BR15686	BR15686		**-**	KJ524309	**-**	Ertz et al. 2015
***M. centrica* sp. nov**.	**HN20171020-1**	**HMAS–L 152060**	**China**	**OR176873**	**OR176540**	**-**	**This study**
***M. centrica* sp. nov**.	**HN20171030-1**	**HMAS–L 152118**	**China**	**OR176874**	**-**	**OR176696**	**This study**
***M. centrica* sp. nov**.	**HN20171045-1**	**HMAS–L 152061**	**China**	**OR176875**	**-**	**-**	**This study**
***M. centrica* sp. nov**.	**HN20171083-2**	**HMAS–L 152072**	**China**	**OR176876**	**-**	**-**	**This study**
***M. centrica* sp. nov**.	**HN20190573**	**HMAS–L 156368**	**China**	**-**	**OR176541**	**OR176697**	**This study**
***M. centrica* sp. nov**.	**HN20192600-2**	**HMAS–L 156504**	**China**	**-**	**OR176542**	**-**	**This study**
***M. centrica* sp. nov**.	**HN20192626-1**	**HMAS–L 152220**	**China**	**-**	**OR176543**	**OR176698**	**This study**
***M. centrica* sp. nov**.	**HN20192630-1**	**HMAS–L 152223**	**China**	**OR176877**	**OR176544**	**-**	**This study**
***M. centrica* sp. nov**.	**HN20192641-2**	**HMAS–L 152229**	**China**	**-**	**OR176545**	**OR176699**	**This study**
***M. centrica* sp. nov**.	**HN20192650-1**	**HMAS–L 156351**	**China**	**OR176878**	**OR176546**	**OR176700**	**This study**
***M. centrica* sp. nov**.	**HN20192652-1**	**HMAS–L 153590**	**China**	**-**	**OR176547**	**OR176701**	**This study**
***M. centrica* sp. nov**.	**HN20192664-1**	**HMAS–L 152236**	**China**	**OR176879**	**OR176548**	**OR176702**	**This study**
***M. centrica* sp. nov**.	**HN20192671-1**	**HMAS–L 152240**	**China**	**-**	**OR176549**	**OR176703**	**This study**
***M. centrica* sp. nov**.	**HN20192697-1**	**HMAS–L 152243**	**China**	**OR176880**	**OR176550**	**OR176704**	**This study**
***M. centrica* sp. nov**.	**HN20192706-1**	**HMAS–L 153593**	**China**	**OR176881**	**-**	**OR176705**	**This study**
***M. centrica* sp. nov**.	**HN20192707-1**	**HMAS–L 152248**	**China**	**-**	**OR176551**	**OR176706**	**This study**
***M. centrica* sp. nov**.	**HN20192708-2**	**HMAS–L 152249**	**China**	**-**	**OR176552**	**OR176707**	**This study**
***M. centrica* sp. nov**.	**HN20192710-2**	**HMAS–L 156352**	**China**	**OR176882**	**OR176553**	**OR176708**	**This study**
***M. centrica* sp. nov**.	**HN20192720-1**	**HMAS–L 153596**	**China**	**-**	**OR176554**	**OR176709**	**This study**
***M. centrica* sp. nov**.	**HN20192730-1**	**HMAS–L 152259**	**China**	**-**	**OR176555**	**-**	**This study**
***M. centrica* sp. nov**.	**HN20192731-1**	**HMAS–L 152260**	**China**	**OR176883**	**-**	**OR176710**	**This study**
***M. centrica* sp. nov**.	**HN20192733-1**	**HMAS–L 152261**	**China**	**-**	**-**	**OR176711**	**This study**
***M. centrica* sp. nov**.	**HN20192734-2**	**HMAS–L 153599**	**China**	**-**	**OR176556**	**OR176712**	**This study**
***M. centrica* sp. nov**.	**HN20192736-1**	**HMAS–L 147683**	**China**	**-**	**-**	**OR176713**	**This stud**y
***M. centrica* sp. nov**.	**HN20192737-1**	**HMAS–L 152262**	**China**	**OR176884**	**-**	**OR176714**	**This study**
***M. centrica* sp. nov**.	**HN20192753-1**	**HMAS–L 152268**	**China**	**-**	**OR176557**	**OR176715**	**This study**
***M. centrica* sp. nov**.	**HN20192754-1**	**HMAS–L 152269**	**China**	**-**	**OR176558**	**OR176716**	**This study**
***M. centrica* sp. nov**.	**HN20192758-1**	**HMAS–L 152271**	**China**	**OR176885**	**OR176559**	**OR176717**	**This study**
***M. centrica* sp. nov**.	**HN20192759-1**	**HMAS–L 152272**	**China**	**OR176886**	**OR176560**	**OR176718**	**This study**
***M. centrica* sp. nov**.	**HN20192762-1**	**HMAS–L 152274**	**China**	**OR176887**	**OR176561**	**OR176719**	**This study**
***M. centrica* sp. nov**.	**HN20192764-1**	**HMAS–L 153602**	**China**	**-**	**OR176562**	**-**	**This study**
***M. centrica* sp. nov**.	**HN20192767-1**	**HMAS–L 152276**	**China**	**OR176888**	**OR176563**	**OR176720**	**This study**
***M. centrica* sp. nov**.	**HN20192768-1**	**HMAS–L 152277**	**China**	**OR176889**	**OR176564**	**OR176721**	**This study**
***M. centrica* sp. nov**.	**HN20192769-1**	**HMAS–L 152278**	**China**	**OR176890**	**OR176565**	**-**	**This study**
***M. centrica* sp. nov**.	**HN20192773-1**	**HMAS–L 152280**	**China**	**OR176891**	**OR176566**	**OR176722**	**This study**
***M. centrica* sp. nov**.	**HN20192781-1**	**HMAS–L 153605**	**China**	**OR176892**	**OR176567**	**OR176723**	**This study**
***M. centrica* sp. nov**.	**HN20192788-1**	**HMAS–L 153512**	**China**	**OR176893**	**OR176568**	**OR176724**	**This study**
***M. centrica* sp. nov**.	**HN20192823-1**	**HMAS–L 152288**	**China**	**OR176894**	**OR176569**	**OR176725**	**This study**
***M. centrica* sp. nov**.	**HN20192824-1**	**HMAS–L 152289**	**China**	**OR176895**	**-**	**OR176726**	**This study**
***M. centrica* sp. nov**.	**HN20192832-1**	**HMAS–L 152293**	**China**	**OR176896**	**OR176570**	**OR176727**	**This study**
***M. centrica* sp. nov**.	**HN20192834-1**	**HMAS–L 153530**	**China**	**OR176897**	**OR176571**	**OR176728**	**This study**
***M. centrica* sp. nov**.	**HN20192837-1**	**HMAS–L 152294**	**China**	**OR176898**	**OR176572**	**OR176729**	**This study**
***M. dimorphoverrucosa* sp. nov**.	**HN20192792-1**	**HMAS–L 147850**	**China**	**OR176899**	**OR176573**	**OR176730**	**This study**
***M. dimorphoverrucosa* sp. nov**.	**HN20192797-3**	**HMAS–L 147851**	**China**	**OR176900**	**OR176574**	**OR176731**	**This study**
***M. dimorphoverrucosa* sp. nov**.	**HN20192801-1**	**HMAS–L 147853**	**China**	**OR176901**	**OR176575**	**OR176732**	**This study**
***M. dimorphoverrucosa* sp. nov**.	**HN20192820-1**	**HMAS–L 147852**	**China**	**-**	**OR176576**	**OR176733**	**This study**
***M. dimorphoverrucosa* sp. nov**.	**HN20192821-1**	**HMAS–L 153521**	**China**	**-**	**OR176577**	**OR176734**	**This study**
***M. dimorphoverrucosa* sp. nov**.	**HN20192853-2**	**HMAS–L 147854**	**China**	**-**	**OR176578**	**OR176735**	**This study**
***M. dispersa***	**Aptroot s.n**.	**B600300083**	**Brasil**	**OR176907**	**OR176584**	**OR176737**	**This study**
***M. dispersa***	**Aptroot s.n**.	**B600300087**	**Brasil**	**OR176908**	**OR176585**	**OR176738**	**This study**
***M. dispersa***	**Aptroot s.n**.	**B600300096**	**Brasil**	**OR176909**	**OR176586**	**OR176739**	**This study**
***M. dispersa***	**Aptroot s.n**.	**B600300103**	**Brasil**	**OR176902**	**OR176579**	**-**	**This study**
***M. dispersa***	**Aptroot s.n**.	**B600300135**	**Brasil**	**OR176903**	**OR176580**	**-**	**This study**
***M. dispersa***	**Aptroot s.n**.	**B600300145**	**Brasil**	**OR176904**	**OR176581**	**OR176736**	**This study**
***M. dispersa***	**Aptroot s.n**.	**B600300222**	**Brasil**	**OR176905**	**OR176582**	**-**	**This study**
***M. dispersa***	**Aptroot s.n**.	**B600300225**	**Brasil**	**OR176906**	**OR176583**	**-**	**This study**
***M. gelatinospora* sp. nov**.	**GD20190964-1**	**HMAS–L 156372**	**China**	**-**	**-**	**OR176740**	**This study**
***M. gelatinospora* sp. nov**.	**GD20190965-1**	**HMAS–L 156373**	**China**	**-**	**-**	**OR176741**	**This study**
***M. gelatinospora* sp. nov**.	**GD20190966-1**	**HMAS–L 156374**	**China**	**-**	**-**	**OR176742**	**This study**
***M. gelatinospora* sp. nov**.	**HN20192655-1**	**HMAS–L 147859**	**China**	**-**	**-**	**OR176743**	**This study**
***M. gelatinospora* sp. nov**.	**HN20192739-1**	**HMAS–L 147858**	**China**	**-**	**-**	**OR176744**	**This study**
***M. gelatinospora* sp. nov**.	**HN20192749-1**	**HMAS–L 152266**	**China**	**-**	**-**	**OR176745**	**This study**
***M. gelatinospora* sp. nov**.	**HN20192833-1**	**HMAS–L 147856**	**China**	**-**	**-**	**OR176746**	**This study**
***M. gelatinospora* sp. nov**.	**HN20192836-2**	**HMAS–L 147857**	**China**	**-**	**-**	**OR176747**	**This study**
***M. gelatinospora* sp. nov**.	**HN20192840-2**	**HMAS–L 147855**	**China**	**-**	**-**	**OR176748**	**This study**
***M. gelatinospora* sp. nov**.	**HN20192856-2**	**HMAS–L 147860**	**China**	**-**	**-**	**OR176749**	**This study**
***M. hainanensis***	**HN20171141-1**	**HMAS–L 153543**	**China**	**-**	**-**	**OR176750**	**This study**
***M. hainanensis***	**HN20171229-1**	**HMAS–L 153547**	**China**	**-**	**-**	**OR176751**	**This study**
***M. hainanensis***	**HN20171263-1**	**HMAS–L 153548**	**China**	**-**	**-**	**OR176752**	**This study**
***M. hainanensis***	**HN20171342-1**	**HMAS–L 153488**	**China**	**-**	**-**	**OR176753**	**This study**
***M. hainanensis***	**HN20171814-2**	**HMAS–L 153500**	**China**	**-**	**-**	**OR176754**	**This study**
***M. hainanensis***	**HN20192501-1**	**HMAS–L 153503**	**China**	**-**	**-**	**OR176755**	**This study**
***M. hainanensis***	**HN20192502-1**	**HMAS–L 153504**	**China**	**-**	**-**	**OR176756**	**This study**
***M. hainanensis***	**HN20192655-2**	**HMAS–L 147859**	**China**	**-**	**-**	**OR176757**	**This study**
***M. hainanensis***	**HN20192656-1**	**HMAS–L 153510**	**China**	**-**	**-**	**OR176758**	**This study**
***M. hainanensis***	**HN20192815-1**	**HMAS–L 153523**	**China**	**-**	**-**	**OR176759**	**This study**
***M. hainanensis***	**HN20192827-1**	**HMAS–L 153528**	**China**	**-**	**-**	**OR176760**	**This study**
***M. hainanensis***	**HN20192834-2**	**HMAS–L 153530**	**China**	**-**	**-**	**OR176761**	**This study**
***M. hainanensis***	**HN20192838-1**	**HMAS–L 153571**	**China**	**-**	**-**	**OR176762**	**This study**
***M. hainanensis***	**HN20192842-1**	**HMAS–L 153532**	**China**	**-**	**-**	**OR176763**	**This study**
***M. hainanensis***	**HN20192845-1**	**HMAS–L 153533**	**China**	**-**	**-**	**OR176764**	**This study**
***M. intermedia* sp. nov**.	**HN20171297-2**	**HMAS–L 152081**	**China**	**-**	**-**	**OR176765**	**This study**
***M. intermedia* sp. nov**.	**HN20192651-1**	**HMAS–L 153589**	**China**	**-**	**OR176587**	**OR176766**	**This study**
***M. intermedia* sp. nov**.	**HN20192757-1**	**HMAS–L 152270**	**China**	**-**	**OR176588**	**OR176767**	**This study**
***M. intermedia* sp. nov**.	**HN20192816-1**	**HMAS–L 152287**	**China**	**-**	**OR176589**	**OR176768**	**This study**
***M. melanophthalma***	**Aptroot s.n**.	**B600300080**	**Brasil**	**OR176915**	**OR176596**	**OR176772**	**This study**
***M. melanophthalma***	**Aptroot s.n**.	**B600300106**	**Brasil**	**-**	**OR176590**	**OR176769**	**This study**
***M. melanophthalma***	**Aptroot s.n**.	**B600300211**	**Brasil**	**OR176910**	**OR176591**	**OR176770**	**This study**
***M. melanophthalma***	**Aptroot s.n**.	**B600300218-1**	**Brasil**	**OR176911**	**OR176592**	**-**	**This study**
***M. melanophthalma***	**Aptroot s.n**.	**B600300221**	**Brasil**	**OR176912**	**OR176593**	**-**	**This study**
***M. melanophthalma***	**Aptroot s.n**.	**B600300224**	**Brasil**	**OR176913**	**OR176594**	**-**	**This study**
***M. melanophthalma***	**Aptroot s.n**.	**B600300230**	**Brasil**	**OR176914**	**OR176595**	**OR176771**	**This study**
*M. melanophthalma*	UPS3b2	UPS 3b2		**-**	KJ851063	**-**	Frisch et al. 2014
***M. papillosa* sp. nov**.	**HN20170661-2**	**HMAS–L 152054**	**China**	**OR176916**	**OR176597**	**-**	**This study**
***M. papillosa* sp. nov**.	**HN20192547**	**HMAS–L 153578**	**China**	**OR176917**	**OR176598**	**-**	**This study**
***M. papillosa* sp. nov**.	**HN20192612-1**	**HMAS–L 152216**	**China**	**-**	**OR176599**	**-**	**This study**
***M. papillosa* sp. nov**.	**HN20192649-1**	**HMAS–L 152232**	**China**	**OR176918**	**OR176600**	**OR176773**	**This study**
***M. papillosa* sp. nov**.	**HN20192666-2**	**HMAS–L 152237**	**China**	**-**	**OR176601**	**OR176774**	**This study**
***M. papillosa* sp. nov**.	**HN20192684**	**HMAS–L 152242**	**China**	**OR176919**	**OR176602**	**OR176775**	**This study**
***M. papillosa* sp. nov**.	**HN20192766-1**	**HMAS–L 152275**	**China**	**-**	**-**	**OR176776**	**This study**
***M. papillosa* sp. nov**.	**HN20192772-1**	**HMAS–L 152279**	**China**	**OR176920**	**OR176603**	**-**	**This study**
***M. papillosa* sp. nov**.	**HN20192772-2**	**HMAS–L 156508**	**China**	**OR176921**	**OR176604**	**-**	**This study**
***M. papillosa* sp. nov**.	**HN20192777-3**	**HMAS–L 153604**	**China**	**-**	**OR176605**	**-**	**This study**
***M. papillosa* sp. nov**.	**HN20192785-1**	**HMAS–L 153606**	**China**	**-**	**OR176606**	**-**	**This study**
***M. papillosa* sp. nov**.	**HN20192786-1**	**HMAS–L 153607**	**China**	**-**	**OR176607**	**-**	**This study**
***M. papillosa* sp. nov**.	**HN20192865-1**	**HMAS–L 152298**	**China**	**OR176922**	**OR176608**	**OR176777**	**This study**
*M. paupercula*	BR9264	BR 9264		**-**	KJ524310	-	Ertz et al. 2015
***M. phyllosema***	**Aptroot s.n**.	**B600300107**	**Brasil**	**OR176923**	**OR176609**	**OR176778**	**This study**
***M. phyllosema***	**HN20171306-1**	**HMAS–L 153550**	**China**	-	**-**	**OR176779**	**This study**
***M. phyllosema***	**HN20171524-1**	**HMAS–L 153495**	**China**	**-**	**-**	**OR176780**	**This study**
***M. phyllosema***	**HN20171602-1**	**HMAS–L 153496**	**China**	**-**	**-**	**OR176781**	**This study**
***M. phyllosema***	**HN20192500-2**	**HMAS–L 153502**	**China**	**-**	**-**	**OR176782**	**This study**
***M. phyllosema***	**HN20192543-1**	**HMAS–L 147112**	**China**	**-**	**-**	**OR176783**	**This study**
***M. phyllosema***	**HN20192556-1**	**HMAS–L 153505**	**China**	**-**	**-**	**OR176784**	**This study**
***M. phyllosema***	**HN20192558-1**	**HMAS–L 153506**	**China**	**-**	**-**	**OR176785**	**This study**
***M. phyllosema***	**HN20192560-1**	**HMAS–L 147113**	**China**	**-**	**-**	**OR176786**	**This study**
***M. phyllosema***	**HN20192564-1**	**HMAS–L 153507**	**China**	**-**	**-**	**OR176787**	**This study**
***M. phyllosema***	**HN20192827-2**	**HMAS–L 153528**	**China**	**-**	**-**	**OR176788**	**This study**
***M. phyllosema***	**HN20192836-1**	**HMAS–L 156506**	**China**	**-**	**-**	**OR176789**	**This study**
***M. phyllosema***	**HN20192852-1**	**HMAS–L 153537**	**China**	**-**	**-**	**OR176790**	**This study**
***M. phyllosema***	**HN20192853-1**	**HMAS–L 156507**	**China**	**-**	**-**	**OR176791**	**This study**
***M. pilosa***	**Aptroot s.n**.	**B600300136**	**Brasil**	**-**	**-**	**OR176792**	**This study**
***M. pilosa***	**Aptroot s.n**.	**B600300163**	**Brasil**	**-**	**OR176610**	**OR176793**	**This study**
***M. pilosa***	**Aptroot s.n**.	**B600300181**	**Brasil**	**-**	**-**	**OR176794**	**This study**
***M. pilosa***	**Aptroot s.n**.	**B600300182**	**Brasil**	**-**	**-**	**OR176795**	**This study**
***M. pilosa***	**Aptroot s.n**.	**B600300199**	**Brasil**	**-**	**OR176611**	**OR176796**	**This study**
***M. pilosa***	**Aptroot s.n**.	**B600300207**	**Brasil**	**-**	**OR176612**	**OR176797**	**This study**
***M. pilosa***	**Aptroot s.n**.	**B600300210**	**Brasil**	**-**	**-**	**OR176798**	**This study**
***M. praemorsa***	**Aptroot s.n**.	**B600300086**	**Brasil**	**-**	**OR176621**	**OR176809**	**This study**
***M. praemorsa***	**Aptroot s.n**.	**B600300095**	**Brasil**	**OR176931**	**OR176622**	**OR176810**	**This study**
***M. praemorsa***	**Aptroot s.n**.	**B600300097**	**Brasil**	**OR176932**	**OR176623**	**-**	**This study**
***M. praemorsa***	**Aptroot s.n**.	**B600300101**	**Brasil**	**OR176924**	**OR176613**	**OR176799**	**This study**
***M. praemorsa***	**Aptroot s.n**.	**B600300102**	**Brasil**	**OR176925**	**OR176614**	**OR176800**	**This study**
***M. praemorsa***	**Aptroot s.n**.	**B600300108**	**Brasil**	**-**	**OR176615**	**OR176801**	**This study**
***M. praemorsa***	**Aptroot s.n**.	**B600300131**	**Brasil**	**-**	**-**	**OR176802**	**This study**
***M. praemorsa***	**Aptroot s.n**.	**B600300132**	**Brasil**	**OR176926**	**OR176616**	**-**	**This study**
***M. praemorsa***	**Aptroot s.n**.	**B600300137**	**Brasil**	**-**	**OR176617**	**OR176803**	**This study**
***M. praemorsa***	**Aptroot s.n**.	**B600300162**	**Brasil**	**OR176927**	**-**	**OR176804**	**This study**
***M. praemorsa***	**Aptroot s.n**.	**B600300177**	**Brasil**	**OR176928**	**OR176618**	**OR176805**	**This study**
***M. praemorsa***	**Aptroot s.n**.	**B600300180**	**Brasil**	**OR176929**	**OR176619**	**OR176806**	**This study**
***M. praemorsa***	**Aptroot s.n**.	**B600300185**	**Brasil**	**OR176930**	**-**	**OR176807**	**This study**
***M. praemorsa***	**Aptroot s.n**.	**B600300218-2**	**Brasil**	**-**	**OR176620**	**OR176808**	**This study**
***M. pruinata* sp. nov**.	**HN20192668-2**	**HMAS–L 152238**	**China**	**OR176933**	**OR176624**	**OR176811**	**This study**
***M. pruinata* sp. nov**.	**HN20192670-2**	**HMAS–L 152239**	**China**	**-**	**OR176625**	**OR176812**	**This study**
***M. pruinata* sp. nov**.	**HN20192701-1**	**HMAS–L 152246**	**China**	**OR176934**	**OR176626**	**-**	**This study**
***M. pruinata* sp. nov**.	**HN20192719-1**	**HMAS–L 152253**	**China**	**OR176935**	**OR176627**	**-**	**This study**
***M. pruinata* sp. nov**.	**HN20192723-1**	**HMAS–L 152255**	**China**	**OR176936**	**OR176628**	**-**	**This study**
***M. pseudoaptrootii* sp. nov**.	**HN2016001-1**	**HMAS–L 152202**	**China**	**OR176937**	**OR176629**	**OR176813**	**This study**
***M. pseudoaptrootii* sp. nov**.	**HN20170610-2**	**HMAS–L 152051**	**China**	**OR176938**	**OR176630**	**OR176814**	**This study**
***M. pseudoaptrootii* sp. nov**.	**HN20170672-2**	**HMAS–L 153486**	**China**	**OR176939**	**OR176631**	**OR176815**	**This study**
***M. pseudoaptrootii* sp. nov**.	**HN20171025-1**	**HMAS–L 152117**	**China**	**OR176940**	**-**	**OR176816**	**This study**
***M. pseudoaptrootii* sp. nov**.	**HN20192603-1**	**HMAS–L 153580**	**China**	**OR176941**	**-**	**OR176817**	**This study**
***M. pseudoaptrootii* sp. nov**.	**HN20192608-2**	**HMAS–L 153581**	**China**	**OR176942**	**-**	**OR176818**	**This study**
***M. pseudoaptrootii* sp. nov**.	**HN20192610-1**	**HMAS–L 153582**	**China**	**OR176943**	**OR176632**	**OR176819**	**This study**
***M. pseudoaptrootii* sp. nov**.	**HN20192611-1**	**HMAS–L 152215**	**China**	**OR176944**	**OR176633**	**OR176820**	**This study**
***M. pseudoaptrootii* sp. nov**.	**HN20192638-1**	**HMAS–L 152228**	**China**	**OR176945**	**OR176634**	**OR176821**	**This study**
***M. pseudoaptrootii* sp. nov**.	**HN20192642-1**	**HMAS–L 152230**	**China**	**OR176946**	**OR176635**	**OR176822**	**This study**
***M. pseudoaptrootii* sp. nov**.	**HN20192775-1**	**HMAS–L 152281**	**China**	**OR176947**	**OR176636**	**OR176823**	**This study**
***M. pseudocorticola* sp. nov**.	**20192319**	**HMAS–L 153577**	**China**	**-**	**OR176637**	**OR176824**	**This study**
***M. pseudomelanophthalma* sp. nov**.	**HN20171494-1**	**HMAS–L 152092**	**China**	**-**	**-**	**OR176825**	**This study**
***M. pseudomelanophthalma* sp. nov**.	**HN20171620**	**HMAS–L 152093**	**China**	**-**	**-**	**OR176826**	**This study**
***M. pseudomelanophthalma* sp. nov**.	**HN20192797-1**	**HMAS–L 156349**	**China**	**-**	**-**	**OR176827**	**This study**
***M. pseudomelanophthalma* sp. nov**.	**HN20192805-1**	**HMAS–L 152284**	**China**	**-**	**-**	**OR176828**	**This stud**y
***M. pseudomelanophthalma* sp. nov**.	**HN20192805-2**	**HMAS–L 156350**	**China**	**-**	**-**	**OR176829**	**This study**
***M. pseudopapillosa* sp. nov**.	**HN20170691-1**	**HMAS–L 146149**	**China**	**OR176948**	**-**	**-**	**This study**
***M. pseudopapillosa* sp. nov**.	**HN20170692-1**	**HMAS–L 146148**	**China**	**OR176949**	**-**	**-**	**This study**
***M. pseudopilosa* sp. nov**.	**HN20192751-1**	**HMAS–L 152267**	**China**	**OR176950**	**OR176638**	**OR176830**	**This study**
***M. pseudopruinata* sp. nov**.	**HN20192698-1**	**HMAS–L 152244**	**China**	**OR176951**	**OR176639**	**OR176831**	**This study**
***M. pseudopruinata* sp. nov**.	**HN20192700-1**	**HMAS–L 152245**	**China**	**OR176952**	**OR176640**	**-**	**This study**
***M. pseudopruinata* sp. nov**.	**HN20192713-1**	**HMAS–L 152250**	**China**	**OR176953**	**OR176641**	**OR176832**	**This study**
***M. pseudopruinata* sp. nov**.	**HN20192714-2**	**HMAS–L 152250**	**China**	**-**	**OR176642**	**-**	**This study**
***M. pseudopruinata* sp. nov**.	**HN20192724-1**	**HMAS–L 152256**	**China**	**OR176954**	**OR176643**	**-**	**This study**
***M. pseudopruinata* sp. nov**.	**HN20192726-1**	**HMAS–L 152257**	**China**	**OR176955**	**OR176644**	**-**	**This study**
***M. pseudopruinata* sp. nov**.	**HN20192728-1**	**HMAS–L 153597**	**China**	**OR176956**	**OR176645**	**OR176833**	**This study**
***M. pseudopruinata* sp. nov**.	**HN20192729-1**	**HMAS–L 152258**	**China**	**OR176957**	**OR176646**	**-**	**This study**
***M. pseudopruinata* sp. nov**.	**HN20192757-2**	**HMAS–L 156346**	**China**	**OR176958**	**OR176647**	**-**	**This study**
***M. pseudopruinata* sp. nov**.	**HN20192842-6**	**HMAS–L 153532**	**China**	**OR176959**	**OR176648**	**OR176834**	**This study**
***M. pseudopruinata* sp. nov**.	**HN20192849-3**	**HMAS–L 153609**	**China**	**-**	**OR176649**	**-**	**This study**
***M. pseudopruinata* sp. nov**.	**HN20192850-1**	**HMAS–L 153574**	**China**	**-**	**OR176650**	**-**	**This study**
***M. pseudopruinata* sp. nov**.	**HN20192861-2**	**HMAS–L 153611**	**China**	**-**	**OR176651**	**-**	**This study**
***M. pseudotenuissima* sp. nov**.	**HN20171090-1**	**HMAS–L 153542**	**China**	**OR176960**	**OR176652**	**-**	**This study**
***M. pseudotenuissima* sp. nov**.	**HN20192600-1**	**HMAS–L 147861**	**China**	**OR176961**	**OR176653**	**-**	**This study**
***M. pseudotenuissima* sp. nov**.	**HN20192643-1**	**HMAS–L 147863**	**China**	**OR176962**	**OR176654**	**-**	**This study**
***M. pseudotenuissima* sp. nov**.	**HN20192644-1**	**HMAS–L 153587**	**China**	**OR176963**	**-**	**-**	**This study**
***M. pseudotenuissima* sp. nov**.	**HN20192650-2**	**HMAS–L 147862**	**China**	**OR176964**	**OR176655**	**OR176835**	**This study**
***M. pseudotenuissima* sp. nov**.	**HN20192710-3**	**HMAS–L 147864**	**China**	**OR176965**	**OR176656**	**OR176836**	**This study**
***M. pseudotenuissima* sp. nov**.	**HN20192712-1**	**HMAS–L 153595**	**China**	**OR176966**	**-**	**-**	**This study**
***M. pseudotenuissima* sp. nov**.	**HN20192868-1**	**HMAS–L 153539**	**China**	**OR176967**	**OR176657**	**OR176837**	**This study**
***M. rotula***	**HN20171489-2**	**HMAS–L 152104**	**China**	**OR176969**	**OR176662**	**OR176838**	**This study**
***M. rotula***	**HN20171556-1**	**HMAS–L 152146**	**China**	**OR176970**	**OR176663**	**OR176839**	**This study**
***M. rotula***	**HN20192795-1**	**HMAS–L 152347**	**China**	**OR176971**	**OR176664**	**OR176840**	**This study**
***M. rotula***	**HN20192810-1**	**HMAS–L 153615**	**China**	**OR176972**	**OR176665**	**OR176841**	**This study**
***M. rotula***	**HN20192838-2**	**HMAS–L 153571**	**China**	**OR176973**	**OR176666**	**OR176842**	**This study**
***M. rotula***	**HN20192842-7**	**HMAS–L 153532**	**China**	**OR176974**	**OR176667**	**OR176843**	**This study**
***M. rotula***	**HN20192862-1**	**HMAS–L 153612**	**China**	**OR176975**	**OR176668**	**OR176844**	**This stud**y
***M. rotula***	**HN20192863-1**	**HMAS–L 153613**	**China**	**OR176976**	**OR176669**	**OR176845**	**This study**
***M. rotula***	**Aptroot s.n**.	**B600300109**	**Brasil**	**-**	**OR176658**	**OR176850**	**This study**
***M. rotula***	**Aptroot s.n**.	**B600300152**	**Brasil**	**OR176968**	**OR176659**	**OR176851**	**This study**
***M. rotula***	**Aptroot s.n**.	**B600300155**	**Brasil**	**-**	**OR176660**	**-**	**This study**
***M. rotula***	**Aptroot s.n**.	**B600300179**	**Brasil**	**-**	**OR176661**	**-**	**This study**
***M. rubropunctata***	**Aptroot s.n**.	**B600300079**	**Brasil**	**-**	**-**	**OR176848**	**This study**
***M. rubropunctata***	**Aptroot s.n**.	**B600300082**	**Brasil**	**-**	**OR176671**	**OR176849**	**This study**
***M. rubropunctata***	**Aptroot s.n**.	**B600300138**	**Brasil**	**OR176977**	**OR176670**	**OR176846**	**This study**
***M. rubropunctata***	**Aptroot s.n**.	**B600300186**	**Brasil**	**OR176978**	**-**	**OR176847**	**This study**
***M. sinensis* sp. nov**.	**HN20192654-1**	**HMAS–L 152234**	**China**	**-**	**-**	**OR176852**	**This study**
***M. sinensis* sp. nov**.	**HN20192661-1**	**HMAS–L 152235**	**China**	**OR176979**	**-**	**OR176853**	**This study**
***M. sinensis* sp. nov**.	**HN20192711-2**	**HMAS–L 153594**	**China**	**OR176980**	**OR176672**	**OR176854**	**This study**
***M. sinensis* sp. nov**.	**HN20192783-1**	**HMAS–L 152283**	**China**	**OR176981**	**OR176673**	**OR176855**	**This study**
***M. straminea* sp. nov**.	**HN20192871-2**	**HMAS–L 152300**	**China**	**-**	**OR176674**	**OR176856**	**This study**
***M. straminea* sp. nov**.	**HN20192872-1**	**HMAS–L 152301**	**China**	**-**	**OR176675**	**OR176857**	**This study**
***M. tenuissima***	**Aptroot s.n**.	**B600300223**	**Brasil**	**-**	**OR176676**	**OR176858**	**This study**
***M. tumidula***	**Aptroot s.n**.	**B600300064**	**Brasil**	**-**	**OR176679**	**OR176861**	**This study**
***M. tumidula***	**Aptroot s.n**.	**B600300090**	**Brasil**	**OR176983**	**OR176680**	**OR176862**	**This study**
***M. tumidula***	**Aptroot s.n**.	**B600300100**	**Brasil**	**-**	**OR176677**	**OR176859**	**This study**
***M. tumidula***	**Aptroot s.n**.	**B600300164**	**Brasil**	**OR176982**	**OR176678**	**OR176860**	**This study**
***M. verrucosa* sp. nov**.	**HN20171715-1**	**HMAS–L 152101**	**China**	**-**	**-**	**OR176863**	**This study**
***M. verrucosa* sp. nov**.	**HN20192509-1**	**HMAS–L 152204**	**China**	**-**	**OR176681**	**OR176864**	**This study**
***M. verrucosa* sp. nov**.	**HN20192513-1**	**HMAS–L 152207**	**China**	**-**	**OR176682**	**OR176865**	**This study**
***M. verrucosa* sp. nov**.	**HN20192539-1**	**HMAS–L 152209**	**China**	**-**	**-**	**OR176866**	**This study**
***M. verrucosa* sp. nov**.	**HN20192540-1**	**HMAS–L 147806**	**China**	**-**	**-**	**OR176867**	**This study**
***M. verrucosa* sp. nov**.	**HN20192542-1**	**HMAS–L 147806**	**China**	**-**	**OR176683**	**-**	**This study**
***M. weii***	**20190437-4**	**HMAS–L 146703**	**China**	**-**	**OR176684**	**OR176868**	**This study**
***M. weii***	**20190565**	**HMAS–L 146702**	**China**	**OR176984**	**OR176685**	**OR176869**	**This study**

Newly generated sequences in this study are shown in bold.

#### Sequence alignment and phylogenetic analyses

Sequences generated from different loci were analyzed with other sequences obtained from GenBank (Table 1). In this study, the genus *Dichosporidium*, which is closely related to *Mazosia*, was selected as the outgroup (Ertz et al. 2015), with *Dichosporidium nigrocinctum* serving as the outgroup reference. Each marker was first aligned independently with MAFFT v. 7.402 (Katoh and Standley 2013) and the combinability was tested (Kauff and Lutzoni 2002). To that end, each marker was first analyzed separately using MrBayes. The Markov chain Monte Carlo algorithm of MrBayes ran with 20 chains simultaneously, each initiated with a random tree, for 2,000,000 generations, sampling every 20^th^ generation for a total of 100,000 trees sampled. The first 4,500 sampled trees were discarded before calculating the majority-rule consensus tree, to ensure that all chains had converged at a single level. A majority-rule consensus tree was calculated with DendroPy v. 4.5.2 for the remaining 95,500 B/MCMC sampled trees (Sukumaran and Mark 2010). The majority-rule consensus tree of each gene was constructed through SumTrees with the parameters “summary-target = consensus –burnin = 4500 –support-as-labels –min-clade-freq = 0.1”. Conflict was assumed to be significant if different relationships for the same set of taxa (one being monophyletic and the other being nonmonophyletic), all with posterior probabilities (PP) 0.99, were observed on the majority-rule consensus trees. Analyses of the single markers and the combined data set resulted in basically the same topology. Since no significant conflict was detected throughout the majority-rule consensus trees, the three markers were concatenated (Kauff and Lutzoni 2002).

An ML tree involving 1000 pseudoreplicates was generated by IQ-TREE v. 1.6.6 (Nguyen et al. 2015), using the concatenated set of the three markers (ITS, LSU, and mtSSU; Suppl. material 1). For this analysis, the best-fit substitution model was selected using ModelFinder (Kalyaanamoorthy et al. 2017), which identifies optimal model of sequence evolution (SE) by combining substitution models (e.g., GTR) with flexible rate heterogeneity across site models. By allowing the tree topology to vary during the search for an optimal model of SE, ModelFinder reduces the chance of entrapment in local optima during model selection. TIMe+I+G4 was selected as the best model in the *Mazosia* analysis.

Bayesian analysis was performed with MrBAYES, assuming the general time reversible model including estimation of invariant sites and a discrete gamma distribution with six rate categories (GTR+I+G), for the single-genes and the combined analyses (Ronquist et al. 2012). A run with 5,000,000 generations and employing 20 simultaneous chains was executed. Posterior probabilities above 95% and bootstrap support above 70% are considered significant supports.

Phylogenetic trees were drawn using FigTree v. 1.4.2 (Rambaut 2012). Sequences derived in this study are deposited in GenBank (https://www.ncbi.nlm.nih.gov), and taxonomic novelties in MycoBank (www.MycoBank.org; Crous et al. 2004).

## Results and discussion

**Figure 1.**
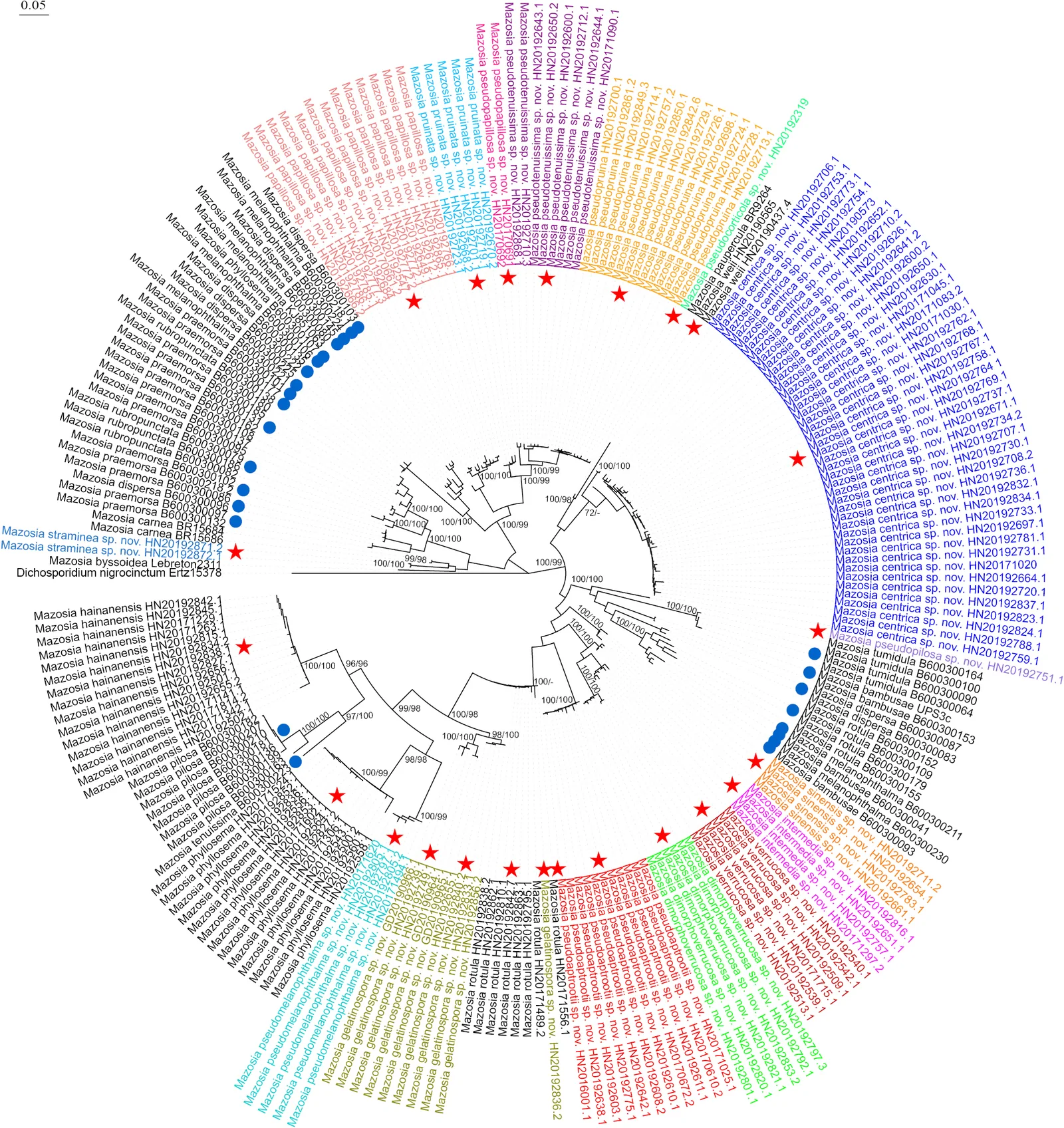
Phylogenetic tree constructed through ML analyses based on ITS, nuLSU, and mtSSU sequences with an alignment length of 2438 bp. Branches are annotated with bootstrap values above 70% (left) and Bayesian inference posterior probabilities above 95% (right), denoted as ML–BS / B–PP. Sixteen novel species are highlighted with distinct colors. The symbols five-pointed star in red and circle in blue designate specimens originating from China and Brazil, respectively. The scale bar located in the upper left indicates 0.1 nucleotide substitutions per site.

The dataset includes 115 ITS sequences, 148 LSU sequences, and 177 mtSSU sequences newly generated for this study. Our multi-locus phylogenetic analysis indicates a high level of previously unrecognized diversity in the genus *Mazosia* (Fig. 1), although the bulk of the sampling was limited to two larger tropical regions, Brazil and China. Overall, we distinguish 48 lineages, 45 corresponding to foliicolous and three to corticolous representatives. Nominally, the 45 foliicolous lineages would have been identified as 15 taxa, employing the current phenotype-based taxonomy focusing on thallus morphology and ascospore septation (Santesson 1952; Lücking 2008). This suggests a threefold increase in the number of species, but this pattern is not equally distributed among the traditionally recognized taxa. While some well-defined taxa, such as *M. tumidula*, formed well-delimited clades, others resulted in polyphyletic species, often with broad geographic separation (e.g., *M. phyllosema*, *M. rotula*) or exhibited pronounced internal diversification, indicating species complexes, such as *M. melanophthalma*, *M. praemorsa*, and *M. tenuissima*, two of these in two geographically separated clades (Fig. 1). Particularly in the cases of *M. melanophthalma* s.lat. and *M. tenuissima* s.lat., the subclades were found to be correlated with subtle phenotypic differences, such as ascospore size or the presence of a gelatinous halo (see taxonomic part below).

The discovery of previously unrecognized diversity in the foliicolous lichenized genus *Mazosia*, with an up to 200% increase in certain lineages, is striking but not surprising. *Mazosia* is one of the most common foliicolous genera, which is regularly present in high individual numbers in closed rainforest understory communities throughout the tropics (Lücking 1999, 2008). Other foliicolous lichens that have recently been assessed using molecular methods also showed high levels of hidden diversity, even more so than *Mazosia*, such as in the genus *Strigula* and its relatives (Jiang et al. 2020, 2022). The presumably pantropical taxon, *S. smaragdula* s.lat., includes numerous, in part unrelated species in just one region of (sub-)tropical Asia (Jiang et al. 2016, 2017, 2022; Woo et al. 2020).

Our integrative multi-locus phylogeny reveals an extraordinary, previously underestimated cryptic radiation of *Mazosia* on Hainan Island—a striking signal that this insular tropical hotspot harbours unique foliicolous lichen diversity unrivalled within continental Southeast China (Wang et al. 2024). Hainan’s isolated island niche, separated from the Chinese mainland by the Qiongzhou Strait, sustains continuous closed-canopy rainforest understories with stable high humidity, uniform mild temperature and abundant evergreen leaf substrates, forming optimal microhabitats for niche partitioning of leaf-dwelling *Roccellaceae* fungi (Zhu et al. 2022). Unlike seasonal subtropical forests of Yunnan and Guangxi, Hainan’s year-round moist microclimate eliminates seasonal desiccation stress, enabling fine-scale ecological differentiation among sympatric *Mazosia* lineages through photobiont selectivity, ascospore morphology and thallus texture variation (Lücking 2008).

Oceanic and tropical island systems repeatedly demonstrate accelerated cryptic speciation in foliicolous lichens via geographic isolation and micro-niche filtering, as documented for Pacific tropical island leaf lichen floras (Sérusiaux and Lücking 2007). Our discovery of 16 novel taxa confirms Hainan functions as an independent evolutionary refugium for foliicolous *Mazosia*, where prolonged geographic seclusion drove in-situ diversification distinct from continental congeners distributed across Indochina and Malesia. The updated global identification key provided here addresses major taxonomic ambiguity caused by rampant morphological convergence, laying a baseline for future biogeographic comparisons between Hainan’s insular lichen biota and other tropical island archipelagos. Further comprehensive surveys across lesser-explored montane rainforest fragments on Hainan are predicted to uncover additional unrecognised *Mazosia* lineages, highlighting the urgent need for targeted conservation of intact understory leaf substrate habitats on this biodiversity-rich tropical island.

### Taxonomy

#### Mazosia centrica

Taxon classificationAnimaliaArthonialesRoccellaceae

S.H. Jiang, Z.T. Yao, J.C. Wei & Lücking sp. nov.

E8BE96B2-B8B8-5097-9F2D-3C24E50B94DD

852568

Fig. 2

##### Etymology.

The epithet refers to the verrucae often formed in concentric circles.

##### Typus.

CHINA • Hainan Province, Baoting County, Qixianling National Nature Reserve, alt. ca. 471 m, on living leaves, 13 Dec. 2019, Z.T. Yao HN20192837-1 (holotype HMAS–L 152294).

##### Diagnosis.

The new species is most similar to *Mazosia melanophthalma*, but differs by its yellow-green, smaller thallus (1–5 mm vs. 5–15 mm in diam.), concentrically arranged verrucae, smaller ascomata (0.08–0.3 mm vs. 0.3–0.7 mm in diam.), and an unidentified substance detected by TLC.

##### Description.

Thallus foliicolous, continuous, 1–5 mm diam., thin (12.5–17.5 µm), yellow-green; without prothallus; verrucae 0.03–0.08 mm diam., slightly paler than thallus, sometimes in a concentric circle from the center regularly, or sometimes only has a regular arrangement at the edges, pilose or not. Photobiont *Phycopeltis* sp., cells rectangular, 7.5–15 × 3.7–7.5 µm, in radiate plates. Ascomata rounded, 0.08–0.30 mm diam., and 70–90 µm high; disc black, translucent when moistened; margin not sloping outwards. Excipulum dark brown, 12.5–20 µm. Hypothecium pale brown, 10–15 µm. Hymenium colorless, 60–75 µm. Asci clavate, 42–60 × 10–12.5 µm. Ascospores colorless, fusiform, I-, 3-septate, 18–23 × 4.5–5 µm, the second cell is slightly enlarged. Pycnidia low conical, 0.06–0.2 mm diam. Macroconidia bacillar, colorless, non-septate, 5–6 × 2–2.5 µm. Microconidia fusiform-ellipsoid, non-septate, 2–2.5 × 0.5–1 µm.

**Figure 2.**
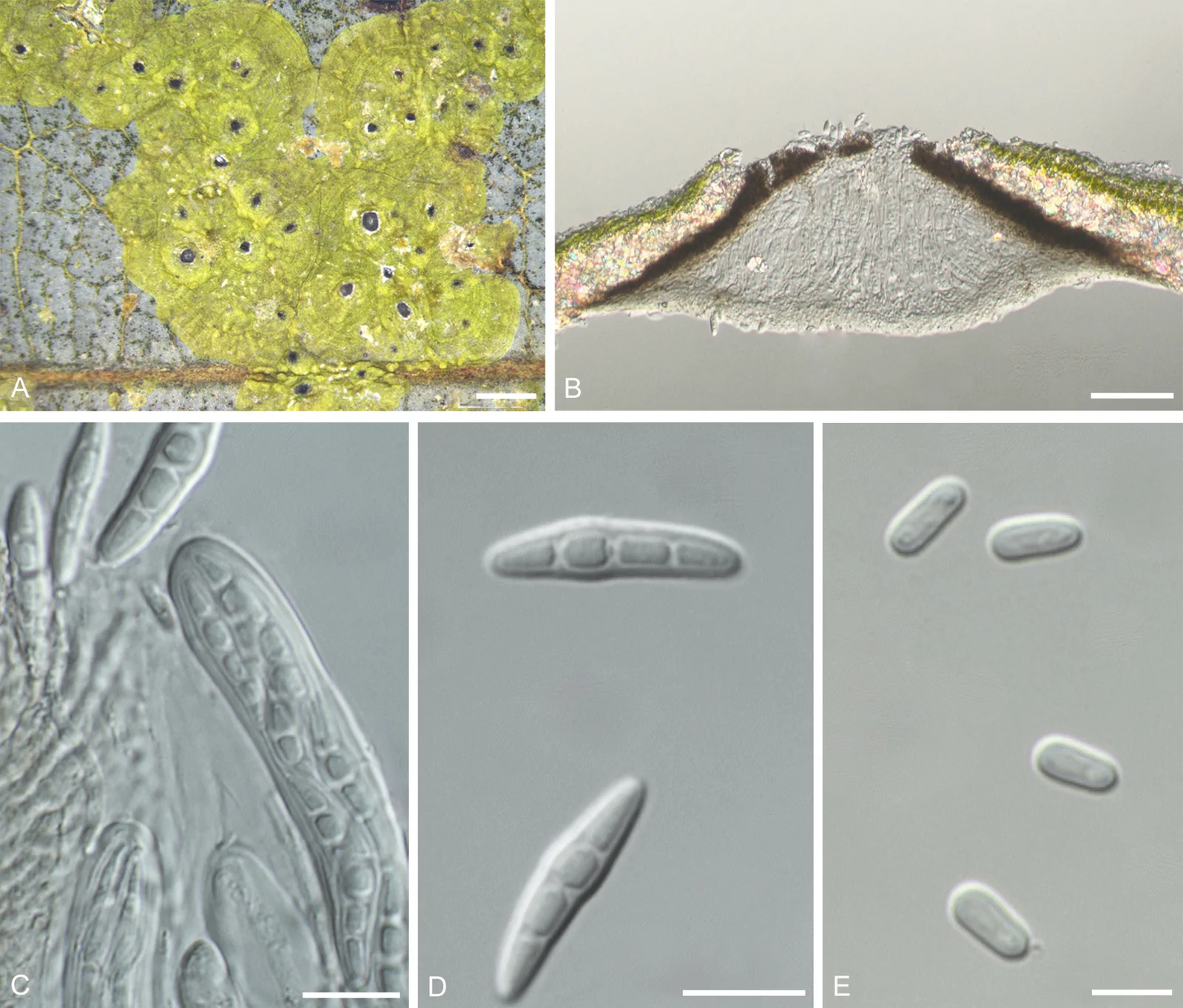
*Mazosia centrica* sp. nov. **A** Thallus with ascomata (HMAS–L 152294). **B** Apothecium section (HMAS–L 152294). **C** Ascus and ascospores (HMAS–L 152294). **D** Ascospores (HMAS–L 152229). **E** Macroconidia (HMAS–L 152060). Scale bars: 0.6 mm (**A**); 50 μm (**B**); 10 μm (**C, D**); 5 μm (**E**).

##### Chemistry.

An unknown substance in region 5–6 in solvent C by TLC.

##### Habitat and distribution.

On unidentified angiosperm leaves in (sub-)tropical regions of China.

##### Additional material examined.

CHINA • Hainan Province, Ledong County, Jianfengling, alt. ca. 960 m, on living leaves, 6 Sep. 2017, S.H. Jiang HN20171018 (HMAS–L 152059), HN20171020-1 (HMAS–L 152060), HN20171030-1 (HMAS–L 152118), HN20171045-1 (HMAS–L 152061), HN20171047-1 (HMAS–L 152124), HN20171054-1 (HMAS–L 152063), HN20171062-1 (HMAS–L 152066), HN20171074-1 (HMAS–L 152069), HN20171076-1 (HMAS–L 152070), HN20171077-1 (HMAS–L 152071), HN20171083-2 (HMAS–L 152072); Mingfenggu, alt. ca. 1002 m, on living leaves, 9 Dec. 2017, X.H. Wu HN17038 (LCUF); alt. ca. 953 m, on living leaves, 9 Sep. 2019, Z.T. Yao HN20192600-2 (HMAS–L 156504), HN20192605-1 (HMAS–L 152213), HN20192626-1 (HMAS–L 152220), HN20192627-1 (HMAS–L 152221), HN20192629-1 (HMAS–L 152222), HN20192630-1 (HMAS–L 152223), HN20192631-1 (HMAS–L 152224); alt. ca. 1019 m, 9 Sep. 2019, Z.T. Yao HN20192634-1 (HMAS–L 152226), HN20192641-2 (HMAS–L 152229), HN20192646 (HMAS–L 152231); alt. ca. 1049 m, 9 Sep. 2019, Z.T. Yao HN20192650-1 (HMAS–L 156351), HN20192652-1 (HMAS–L 153590); alt. ca. 654 m, 10 Sep. 2019, Z.T. Yao HN20192664-1 (HMAS–L 152236), HN20192671-1 (HMAS–L 152240), HN20192676-1 (HMAS–L 153591); alt. ca. 692 m, 10 Sep. 2019, Z.T. Yao HN20192697-1 (HMAS–L 152243), HN20192705-1 (HMAS–L 152247), HN20192706-1 (HMAS–L 153593), HN20192707-1 (HMAS–L 152248), HN20192708-2 (HMAS–L 152249), HN20192710-2 (HMAS–L 156352); alt. ca. 783 m, 10 Sep. 2019, Z.T. Yao HN20192716-3 (HMAS–L 152252), HN20192720-1 (HMAS–L 153596), HN20192731-1 (HMAS–L 152260), HN20192730-1 (HMAS–L 152259), HN20192733-1 (HMAS–L 152261), HN20192734-2 (HMAS–L 153599). Wuzhishan City, Wuzhi Mountain, alt. ca. 800 m, on living leaves, 8 Sep. 2017, S.H. Jiang HN20171924-1 (HMAS–L 153203), HN20172070-1 (HMAS–L 152198); alt. ca. 743 m, on living leaves, 12 Dec. 2019, Z.T. Yao HN20192737-1 (HMAS–L 152262), HN20192753-1 (HMAS–L 152268), HN20192754-1 (HMAS–L 152269), HN20192758-1 (HMAS–L 152271), HN20192759-1 (HMAS–L 152272); alt. ca. 784 m, on living leaves, 12 Dec. 2019, Z.T. Yao HN20192762-1 (HMAS–L 152274), HN20192764-1 (HMAS–L 153602), HN20192767-1 (HMAS–L 152276), HN20192768-1 (HMAS–L 152277), HN20192769-1 (HMAS–L 152278), HN20192771-1 (HMAS–L 153603), HN20192773-1 (HMAS–L 152280), HN20192781-1 (HMAS–L 153605). Baoting County, Qixianling National Nature Reserve, alt. ca. 303 m, on living leaves, 13 Dec. 2019, Z.T. Yao HN20192788-1 (HMAS–L 153512), HN20192789-2 (HMAS–L 153513); alt. ca. 471 m, on living leaves, 13 Dec. 2019, Z.T. Yao HN20192823-1 (HMAS–L 152288), HN20192824-1 (HMAS–L 152289), HN20192828-1 (HMAS–L 152290), HN20192829-1 (HMAS–L 152291), HN20192831-2 (HMAS–L 152292), HN20192832-1 (HMAS–L 152293), HN20192834-1 (HMAS–L 153530), HN20192841-2 (HMAS–L 153531), HN20192843-1 (HMAS–L 152295), HN20192844-1 (HMAS–L 152296), HN20192852-2 (HMAS–L 156505), HN20192855-3 (HMAS–L 153575), HN20192858-1 (HMAS–L 152297), HN20192883-1 (HMAS–L 153614). Guangxi Province, Shangsi County, Shiwan mountain forest park, alt. ca. 290 m, on living leaves, 21 Mar. 2019, S.H. Jiang & C. Zhang 20191030 (HMAS–L 156361); alt. ca. 400 m, 21 Mar. 2019, W.C. Wang 20190573 (HMAS–L 156368). Guangdong Province, Shenzhen City, Fairy Lake Botanical Garden, alt. ca. 65 m, 18 January 2019, F.Y. Liu LCUF GD19401, LCUF GD19394, GD19408 (LCUF), GD19409 (LCUF), GD19388 (LCUF), GD19389 (LCUF), GD19390 (LCUF), GD19391 (LCUF), GD19392 (LCUF), GD19393 (LCUF), GD19395 (LCUF), GD19396 (LCUF), GD19397 (LCUF), GD19398 (LCUF), GD19399 (LCUF), GD19400 (LCUF), GD19402 (LCUF), GD19403 (LCUF), GD19404 (LCUF), GD19405 (LCUF), GD19406 (LCUF), GD19407 (LCUF).

##### Notes.

The main features of this new species are the yellow-green thallus with paler verrucae arranged in concentric circles and an unknown substance detected in region with Rf = 5–6 of system in solvent C by TLC. *Mazosia melanophthalma* is the most similar species, but its thallus is typically pale green and larger (5–15 mm in diam.) and the verrucae are dispersed, the ascomata are larger (0.3–0.7 mm in diam.), and no substances are detected by TLC (Lücking 2008). Other similar species with thallus verrucae are *Mazosia dispersa* and *M. pseudobambusae*. *Mazosia dispersa* often has a pruinose thallus and larger (26–39 × 4–7 µm), (4–)5(–7)-septate ascospores (Lücking 2008), whereas *M. pseudobambusae* differs by its pale brownish grey thallus with a dark prothallus and larger ascospores (20–28 × 4–5 µm; Lücking 2008).

#### Mazosia dimorphoverrucosa

Taxon classificationAnimaliaArthonialesRoccellaceae

S.H. Jiang, Z.T. Yao, J.C. Wei & Lücking sp. nov.

7CB1E037-BD02-52F3-8BC1-5051694D3A29

852569

Fig. 3

##### Etymology.

The epithet refers to the morphological differentiation of the verrucae in the central and peripheral parts of the thallus.

##### Typus.

CHINA • Hainan Province, Baoting County, Qixianling National Nature Reserve, alt. ca. 303 m, on living leaves, 13 Dec. 2019, Z.T. Yao HN20192792-1 (holotype HMAS–L 147850).

##### Diagnosis.

The new species is characterized by two different colors of verrucae, e.g., verrucae in the center of the thallus are often brown and arranged radially, and the verrucae around often with the same color of thallus.

##### Description.

Thallus yellow-green to pale yellow, continuous, marginally rounded, 3–10 mm across, thin (7.5–12.5 µm), surrounded by a dark brown prothallus, verrucose; verrucae in the center of the thallus often brown and arranged radially, towards the periphery of the same color as the thallus and dispersed, 0.03–0.08 mm. Photobiont *Phycopeltis* sp., cells rectangular, 10–12.5 × 5–7.5 µm, in radiate plates. Ascomata rounded, 0.1–0.4 mm diam., and 75–90 µm high; disc dark gray to black, translucent when moistened; margin gently sloping outwards. Excipulum brown, 12.5–20 µm thick. Hypothecium 7.5–12.5 µm high, pale brown. Hymenium 63–75 µm high, colorless. Asci clavate, 38–50 × 10–12.5 µm, 8-spored. Ascospores fusiform, 3-septate, 15–26 × 3.8–5 µm. Pycnidia low conical, 0.1–0.2 mm diam. Macroconidia bacillar, non-septate, 6.5–7.5 × 2–2.5 µm. Microconidia not seen.

**Figure 3.**
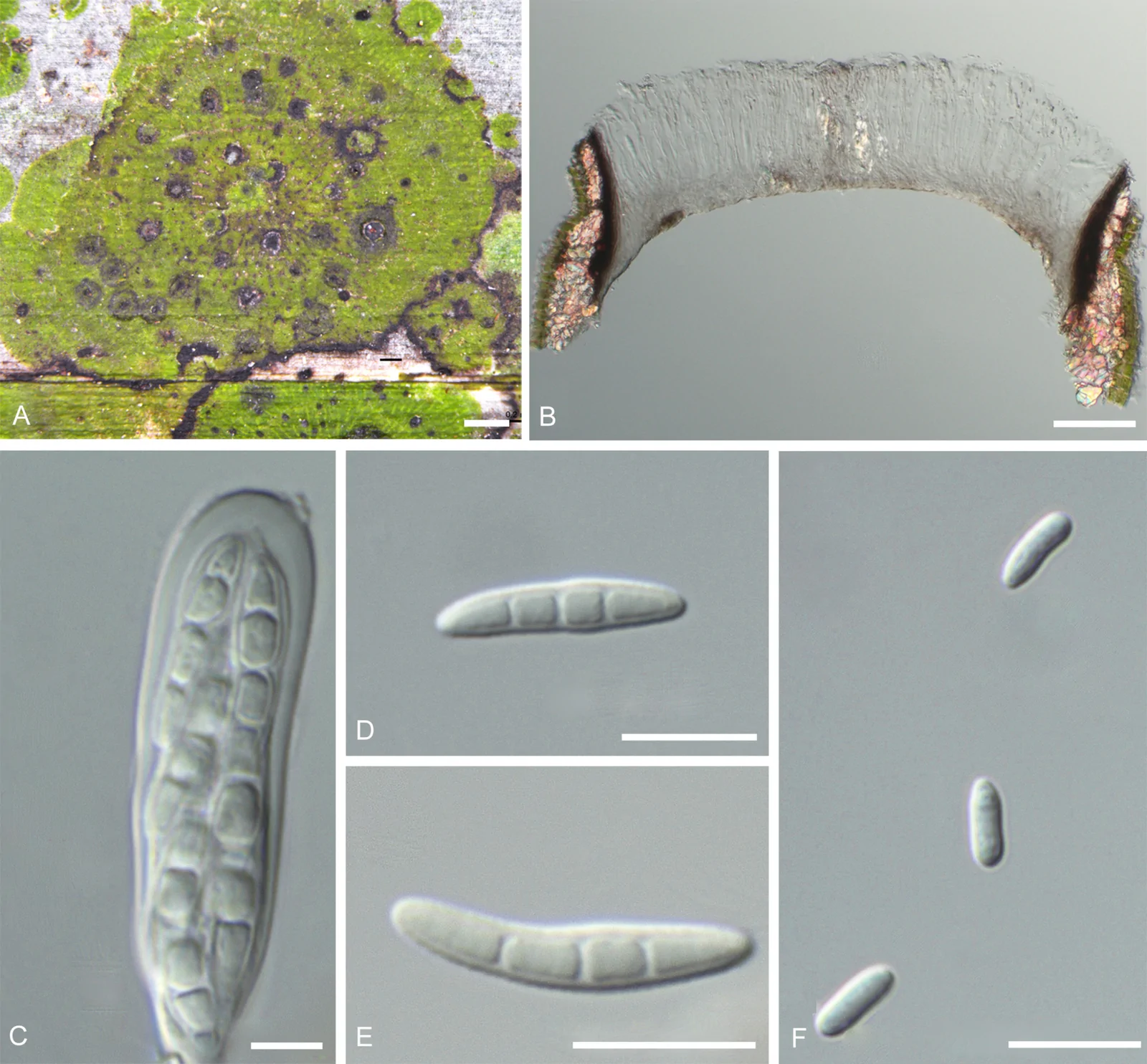
*Mazosia dimorphoverrucosa* sp. nov. **A** Thallus with ascomata (HMAS–L 147850). **B** Apothecium section (HMAS–L 147850). **C** Ascus (HMAS–L 147851). **D, E** Ascospores (HMAS–L 147850). **F** Macroconidia (HMAS–L 147854). Scale bars: 0.4 mm (**A**); 50 μm (**B**); 5 μm (**C**), 10 μm (**D–F**).

##### Chemistry.

No substances detected by TLC.

##### Habitat and distribution.

On unidentified angiosperm leaves in tropics; known only from the type locality, Hainan.

##### Additional material examined.

CHINA • Hainan Province, Baoting County, Qixianling National Nature Reserve, alt. ca. 350 m, on living leaves, 7 Sep. 2017, S.H. Jiang HN20171207-1 (HMAS–L 153545), HN20171259-1 (HMAS–L 153551), HN20171488-2 (HMAS–L 153552), HN20171541-2 (HMAS–L 153555), HN20171560-1 (HMAS–L 153497), HN20171566-1 (HMAS–L 153498), HN20171580-1 (HMAS–L 153499), HN20171612-1 (HMAS–L 153556); alt. ca. 303 m, on living leaves, 13 Dec. 2019, Z.T. Yao HN20192791-1 (HMAS–L 153567), HN20192793-1 (HMAS–L 153568), HN20192794-1 (HMAS–L 153515), HN20192796-1 (HMAS–L 153516), HN20192797-3 (HMAS–L 147851), HN20192799-1 (HMAS–L 153517), HN20192800-1 (HMAS–L 153518), HN20192801-1 (HMAS–L 147853), HN20192803-1 (HMAS–L 153569), HN20192804-1 (HMAS–L 153570), HN20192807-1 (HMAS–L 153519), HN20192817-1 (HMAS–L 153520), HN20192819-2 (HMAS–L 153524), HN20192820-1 (HMAS–L 147852), HN20192821-1 (HMAS–L 153521), HN20192826-1 (HMAS–L 153526); alt. ca. 471 m, on living leaves, 13 Dec. 2019, Z.T. Yao HN20192851-1 (HMAS–L 153536), HN20192853-2 (HMAS–L 147854), HN20192854-1 (HMAS–L 153576).

##### Notes.

The main feature of this new species is that the verrucae in the center of the thallus are often brown and arranged radially, whereas the peripheral verrucae have the same color as the thallus and are dispersed. This appears to be the only species in *Mazosia* with such a variation. The radially arranged verrucae remind of the type species of the genus, *Mazosia rotula*, which differs in having radiate ridges of thallus color or paler and lacking verrucae. Further, *M. rotula* produces psoromic acid and has smaller macroconidia (4–6 × 2–3 µm; Lücking 2008).

#### Mazosia gelatinospora

Taxon classificationAnimaliaArthonialesRoccellaceae

S.H. Jiang, Z.T. Yao, J.C. Wei & Lücking sp. nov.

4A94BEF3-D019-5669-B831-5903E3E060A8

852570

Fig. 4

##### Etymology.

The epithet *gelatinospora* refers to the ascospores being surrounded by a gelatinous sheath.

##### Typus.

CHINA • Hainan Province, Baoting County, Qixianling National Nature Reserve, alt. ca. 471 m, on living leaves, 13 Dec. 2019, Z.T. Yao HN20192840-2 (holotype HMAS–L 147855).

##### Diagnosis.

The new species is distinguishable from other verrucose *Mazosia* species with 3-septate ascospores being surrounded by a gelatinous sheath.

##### Description.

Thallus foliicolous, yellowish green, 4–6 mm across, thin (18–23 µm), with dense verrucae, 0.03–0.08 mm diam., slightly paler than thallus, those on the edge are arranged in concentric circles. Photobiont *Phycopeltis* sp., cells rectangular, 10–23 × 5–10 µm, in radiate plates. Ascomata rounded, 0.1–0.3 mm diam., and 75–115 µm high; disc pale grey black, translucent when moistened; margin gently sloping outwards. Excipulum dark brown, 12.5–17.5 µm thick. Hypothecium pale brown, 20–25 µm high. Hymenium colorless, 55–90 µm high. Asci clavate, 45–55 × 10–15 µm, 8-spored. Ascospores fusiform, 3-septate, 18–25 × 4.5–5 µm, surrounded by a gelatinous sheath, c. 2 µm, the second cell is slightly enlarged. Pycnidia low conical, 0.07–0.15 mm diam. Macroconidia bacillar, non-septate, 5–6.5 × 2–2.5 µm. Microconidia not observed.

**Figure 4.**
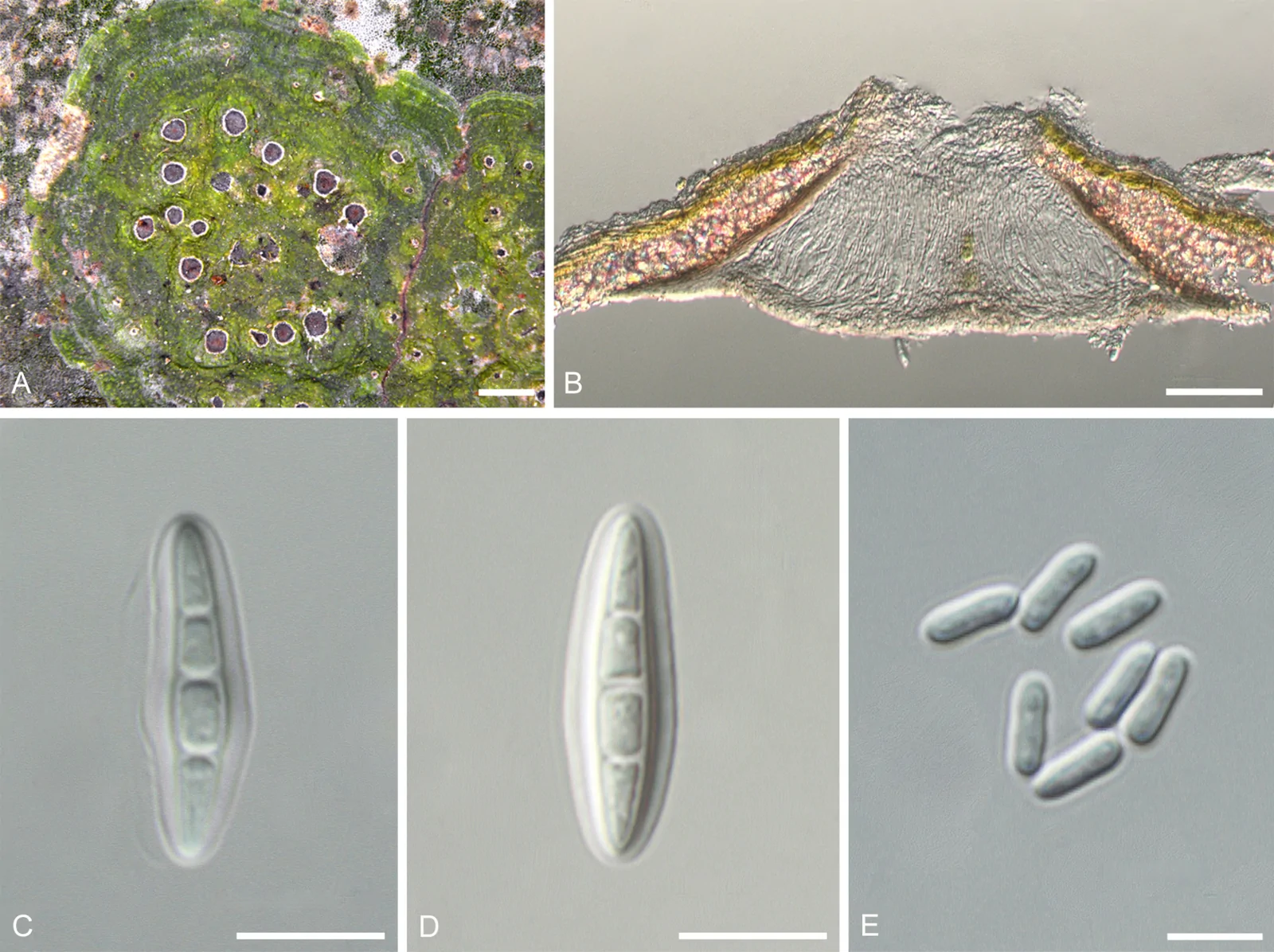
*Mazosia gelatinospora* sp. nov. (HMAS–L 147855). **A** Thallus with ascomata. **B** Apothecium section. **C, D** Ascospores. **E** Macroconidia. Scale bars: 0.6 mm (**A**); 50 μm (**B**); 10 μm (**C, D**), 5 μm (**E**).

##### Chemistry.

No substances detected by TLC.

##### Habitat and distribution.

On unidentified angiosperm leaves in (sub-)tropical regions of China.

##### Additional material examined.

CHINA • Hainan Province, Ledong County, Jianfengling, alt. ca. 1049 m, on living leaves, 9 Dec. 2019, Z.T. Yao HN20192655-1 (HMAS–L 147859). Wuzhishan City, Wuzhi Mountain, alt. ca. 743 m, on living leaves, 12 Dec. 2019, Z.T. Yao HN20192739-1 (HMAS–L 147858), HN20192749-1 (HMAS–L 152266). Baoting County, Qixianling National Nature Reserve, alt. ca. 471 m, on living leaves, 13 Dec. 2019, Z.T. Yao HN20192833-1 (HMAS–L 147856), HN20192836-2 (HMAS–L 147857), HN20192856-2 (HMAS–L 147860). Guangdong Province, Shixing County, Chebaling National Nature Reserve, alt. ca. 420 m, on living leaves, 19 Mar. 2019, S.H. Jiang & C. Zhang 20190962-1 (HMAS–L 156370), 20190963-1 (HMAS–L 156371), 20190964-1 (HMAS–L 156372), 20190965-1 (HMAS–L 156373), 20190966-1 (HMAS–L 156374), 20190969 (HMAS–L 156375).

##### Notes.

*Mazosia gelatinospora* is characterized by a verrucose thallus and 3-septate ascospores, similar to *M. melanophthalma*, but in the new species the ascospores are surrounded by a gelatinous sheath, which has not been observed in other *Mazosia* species. *Mazosia melanophthalma* also has larger ascomata (0.3–0.7 mm) and smaller ascospores (15–22 × 3–5 µm; Lücking 2008).

#### Mazosia intermedia

Taxon classificationAnimaliaArthonialesRoccellaceae

S.H. Jiang, Z.T. Yao, J.C. Wei & Lücking sp. nov.

BD78DD92-53C3-5918-8657-CF5A881AEC00

852571

Fig. 5

##### Etymology.

The epithet refers to the thallus being intermediate between that of *Mazosia pseudobambusae* and *M. melanophthalma*.

##### Typus.

CHINA • Hainan Province, Ledong County, Jianfengling, alt. ca. 1049 m, on living leaves, 9 Dec. 2019, Z.T. Yao HN20192651-1 (holotype HMAS–L 153589).

##### Diagnosis.

The new species is similar to *Mazosia pseudobambusae*, but can be distinguished by narrower ascospores (18–29 × 2.5–3.5 µm vs. 20–28 × 4–5 µm) and macroconidia (4–5 × 1.5–2 µm vs. 4–6 × 2–2.5 µm).

##### Description.

Thallus foliicolous, continuous, yellowish green, slightly shiny, usually rounded, 1.2–2 mm across, thin (9–13 µm), surrounded by a dark brown prothallus, but sometimes incomplete; verrucae 0.03–0.05 mm diam., with the same color of thallus. Photobiont *Phycopeltis* sp., cells rectangular, 10–15 × 2.5–5 µm, in radiate plates. Ascomata rounded, 0.05–0.15 mm diam., and 60–80 µm high; disc black, translucent when moistened; margin gently sloping outwards. Excipulum dark brown, 11–18 µm thick. Hypothecium pale brown, 7–15 µm high. Hymenium colorless, 50–65 µm high. Asci clavate, 47–50 × 10.5–12 µm, 8-spored. Ascospores fusiform, 3-septate, 18–29 × 2.5–3.5 µm, the second cell is slightly enlarged. Pycnidia low conical, 0.02–0.04 mm diam. Macroconidia bacillar, non-septate, 4–5 × 1.5–2 µm; Microconidia not seen.

**Figure 5.**
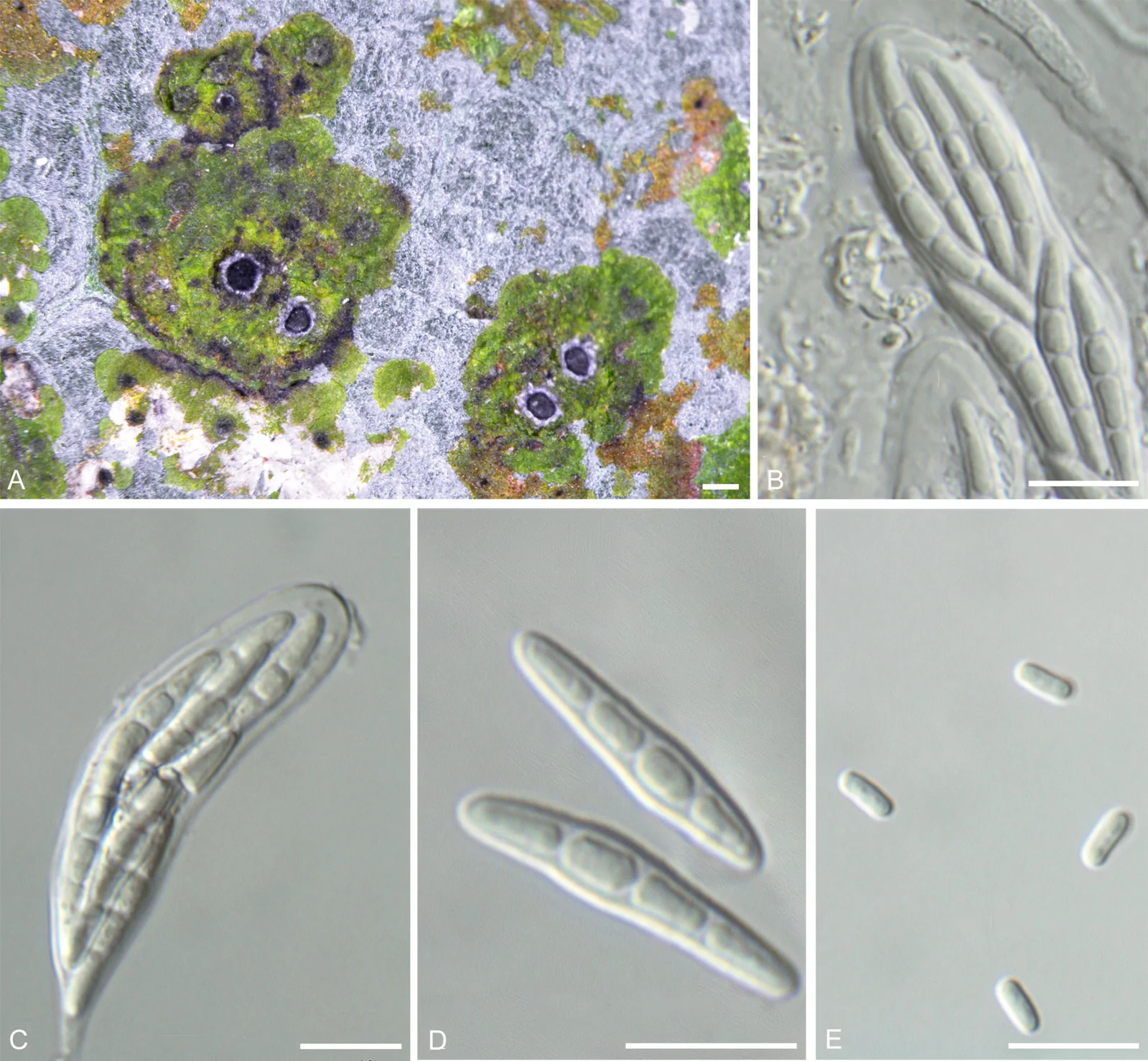
*Mazosia intermedia* sp. nov. (HMAS–L 153589). **A** Thallus with ascomata. **B** Apothecium section. **C** Ascus and ascospores. **D** Ascospores. **E** Macroconidia. Scale bars: 0.2 mm (**A**); 10 μm (**B–E**).

##### Chemistry.

No substances detected by TLC.

##### Ecology and distribution.

On unidentified angiosperm leaves in tropics; known only from the type locality, Hainan.

##### Additional material examined.

CHINA • Hainan Province, Changjiang County, Bawangling National Nature Reserve, alt. ca. 550 m, on living leaves, 5 Sep. 2017, S.H. Jiang HN20171390 (HMAS–L 152087); alt. ca. 700 m, on living leaves, 4 Sep. 2017, S.H. Jiang HN20171734-1 (HMAS–L 152170). Wuzhishan City, Wuzhi Mountain, alt. ca. 743 m, on living leaves, 12 Sep. 2019, Z.T. Yao HN20192757-1 (HMAS–L 152270). Baoting County, Qixianling National Nature Reserve, alt. ca. 303 m, on living leaves, 13 Dec. 2019, Z.T. Yao HN20192816-1 (HMAS–L 152287); alt. ca. 350 m, on living leaves, 7 Sep. 2017, S.H. Jiang HN20171297-2 (HMAS–L 152081).

##### Notes.

The new species is closely related to *Mazosia pseudobambusae*, but the latter has a pale brownish grey thallus, wider ascospores (20–28 × 4–5 µm), and wider macroconidia (4–6 × 2–2.5 µm; Lücking 2008). *Mazosia melanophthalma* differs by larger verrucae (0.05–0.1 mm) on a pale green thallus, smaller ascospores (15–22 × 3–5 µm), and larger macroconidia (5–8 × 2–3 µm; Lücking 2008). *Mazosia verrucosa* (see below) is also similar to the new species, but its thallus is pale yellow-green and usually aggregated in larger groups, without a dark prothallus, and the macroconidia are somewhat larger (5–6.5 × 1.5–2 µm; see below).

#### Mazosia papillosa

Taxon classificationAnimaliaArthonialesRoccellaceae

S.H. Jiang, Z.T. Yao, J.C. Wei & Lücking sp. nov.

BA779A8B-EF6C-5B7E-82F2-98CB711E9E31

852572

Fig. 6

##### Etymology.

The epithet refers to the verrucae of the species appear to be papillary.

##### Typus.

CHINA • Hainan Province, Wuzhishan City, Wuzhi Mountain, alt. ca. 784 m, on living leaves, 12 Dec. 2019, Z.T. Yao HN20192772-1 (holotype HMAS–L 152279).

##### Diagnosis.

The new species is similar to *Mazosia pseudotenuissima*, but can be distinguished by a thallus with a white pruina, smaller verrucae (0.02–0.05 mm vs. 0.04–0.07 mm in diam.), and broader macroconidia (5–6 × 2.2–2.7 µm vs. 4–4.7 × 1.7–2.2 µm).

##### Description.

Thallus foliicolous, continuous, pale yellowish green to pale green, slightly shiny, 2–5 mm across, thin (12.5–20 µm), with white pruina, verrucae, pilose or not; verrucae 0.02–0.05 mm diam., the tops of the verrucae are sometimes whitish, sometimes with the same color of thallus; hairs formed by single, unbranched hyphae. Photobiont *Phycopeltis* sp., cells rectangular, 10–12.5 × 3.8–5 µm, in radiate plates. Ascomata rounded, 0.1–0.3 mm diam., and 100–125 µm high; disc dark gray, translucent when moistened; margin gently sloping outwards. Excipulum dark brown, 17.5–25 µm thick. Hypothecium colorless, 12.5–17.5 µm high. Hymenium colorless, 90–100 µm high. Asci clavate, 38–75 × 10–18 µm, 8-spored. Ascospores fusiform, colorless, 3-septate, 22–33 × 4.5–5.5 µm. Pycnidia low conical, 0.02–0.06 mm diam. Macroconidia bacillar, non-septate, 5–6 × 2.2–2.7 µm; Microconidia not seen.

**Figure 6.**
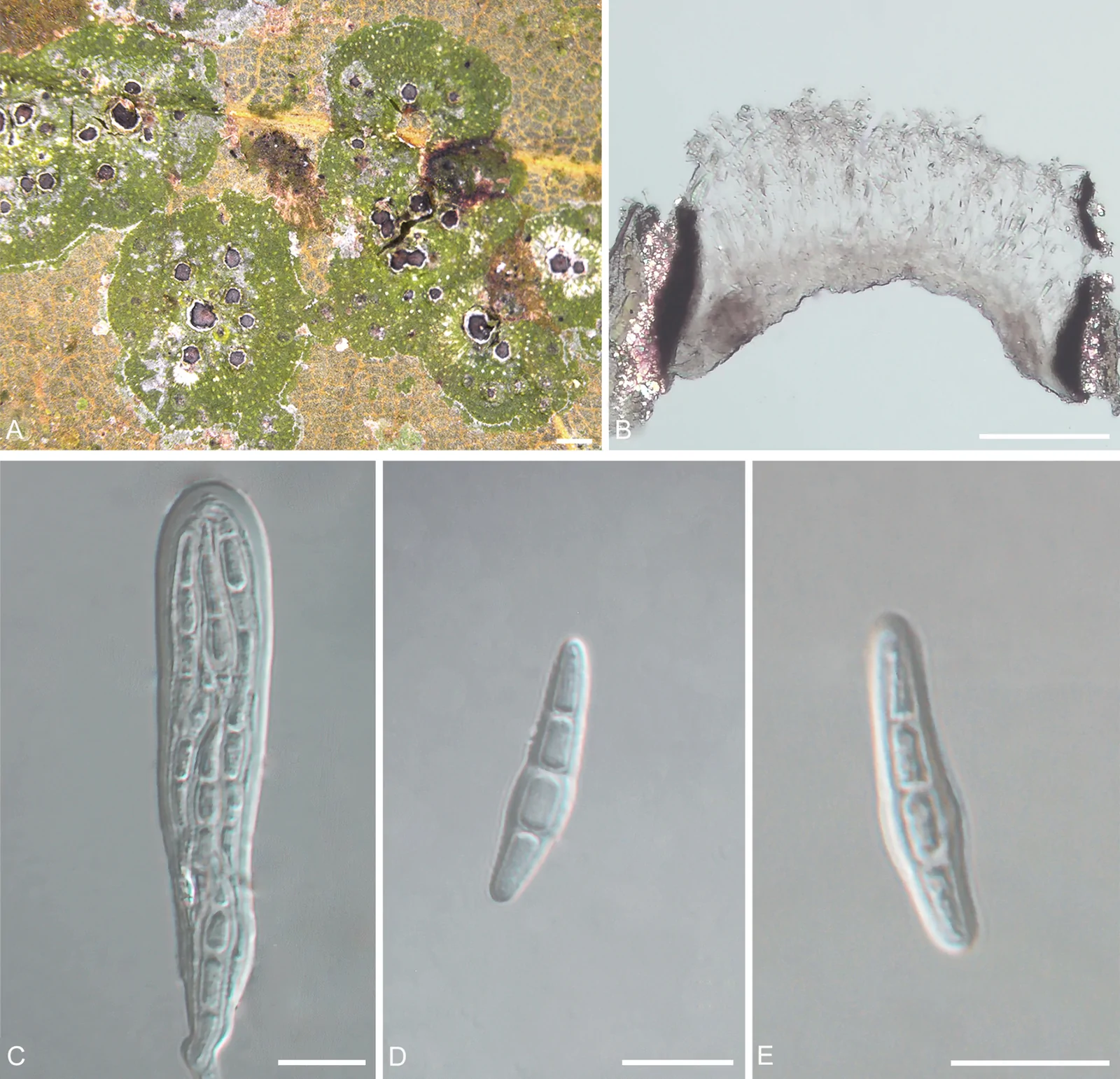
*Mazosia papillosa* sp. nov. (HMAS–L 152279). **A** Thallus with ascomata. **B** Apothecium section. **C** Ascus and ascospores. **D, E** Ascospores. Scale bars: 0.4 mm (**A**); 100 μm (**B**); 10 μm (**C–E**).

##### Chemistry.

No substances detected by TLC.

##### Ecology and distribution.

On unidentified angiosperm leaves in tropics; known only from the type locality, Hainan.

##### Additional material examined.

CHINA • Hainan Province, Qiongzhong County, Limu Mountain National Nature Reserve, alt. ca. 800 m, on living leaves, 10 Sep. 2017, S.H. Jiang HN20170661-2 (HMAS–L 152054), HN20170664-1 (HMAS–L 152055). Bawangling National Nature Reserve, alt. ca. 551 m, on living leaves, 8 Dec. 2019, Z.T. Yao HN20192547 (HMAS–L 153578). Ledong County, Jianfengling, alt. ca. 953 m, on living leaves, 9 Dec. 2019, Z.T. Yao HN20192612-1 (HMAS–L 152216); alt. ca. 1049 m, on living leaves, 9 Dec. 2019, Z.T. Yao HN20192649-1 (HMAS–L 152232); alt. ca. 654 m, on living leaves, 10 Dec. 2019, Z.T. Yao HN20192666-2 (HMAS–L 152237), HN20192684 (HMAS–L 152242). Wuzhishan City, Wuzhi Mountain, alt. ca. 784 m, on living leaves, 12 Dec. 2019, Z.T. Yao HN20192766-1 (HMAS–L 152275), HN20192772-2 (HMAS–L 156508). HN20192777-3 (HMAS–L 153604); alt. ca. 890 m, on living leaves, 12 Dec. 2019, Z.T. Yao HN20192785-1 (HMAS–L 153606), HN20192786-1 (HMAS–L 153607). Lingshui County, Diaoluo Mountain National Nature Reserve, alt. ca. 928 m, on living leaves, 14 Dec. 2019, Z.T. Yao HN20192865-1 (HMAS–L 152298).

##### Notes.

The important features of this species include a thallus with patchily white pruina, verrucae, and 3-septate ascospores (22.5–32.5 × 4.75–5.25 µm). The new species is similar to *Mazosia pseudotenuissima* (see below), but the latter has no pruina, the verrucae are larger (0.04–0.07 mm in diam.) and whitish at the top in the central parts of the thallus, and the macroconidia are narrower (4–4.7 × 1.7–2.2 µm; see below). The new species is related to *Mazosia dispersa*, with which it shares the often pruinose thallus, but the latter can be distinguished by (4–)5(–7)-septate ascospores (Lücking 2008). Another similar species to white pruina is *Mazosia pruinata* (see below), but that taxon differs by the larger verrucae (0.04–0.08 mm diam.), the shorter macroconidia (3.5–5 × 1.5–2 µm), and the pruina is often concentrated in the thallus center (see below).

#### Mazosia pruinata

Taxon classificationAnimaliaArthonialesRoccellaceae

S.H. Jiang, Z.T. Yao, J.C. Wei & Lücking sp. nov.

361B0598-2191-572D-85F1-B3F891044A26

852573

Fig. 7

##### Etymology.

The epithet refers to the thallus often furnished with a white pruina.

##### Typus.

CHINA • Hainan Province, Ledong County, Jianfengling, alt. ca. 783 m, on living leaves, 10 Dec. 2019, Z.T. Yao HN20192723-1 (holotype HMAS–L 152255).

##### Diagnosis.

The new species is characterized by a verrucose and pilose thallus with white pruina and ascospores 3-septate, or occasionally 4–5-septate (25–37 × 4–5 µm).

##### Description.

Thallus foliicolous, continuous, yellowish green, irregular rounded, 1.5–6 mm across, thin (17–20 µm), with white pruina, verrucae, pilose; verrucae 0.04–0.08 mm diam., with the same color of thallus; hairs formed by single, unbranched hyphae. Photobiont *Phycopeltis* sp., cells rectangular, 10–12.5 × 3.8–5 µm, in radiate plates. Ascomata rounded, 0.1–0.22 mm diam., and 80–90 µm high; disc dark gray, translucent when moistened; margin gently sloping outwards. Excipulum dark brown, 8–15 µm thick. Hypothecium colorless, 15–21 µm high. Hymenium colorless, 60–70 µm high. Asci clavate, 39–58 × 12–15 µm, 8-spored. Ascospores fusiform, 3-septate, or occasionally 4–5-septate, 25–37 × 4–5 µm. Pycnidia low conical, 0.25–0.05 mm diam. Macroconidia bacillar, non-septate, 3.5–5 × 1.5–2 µm; Microconidia not seen.

**Figure 7.**
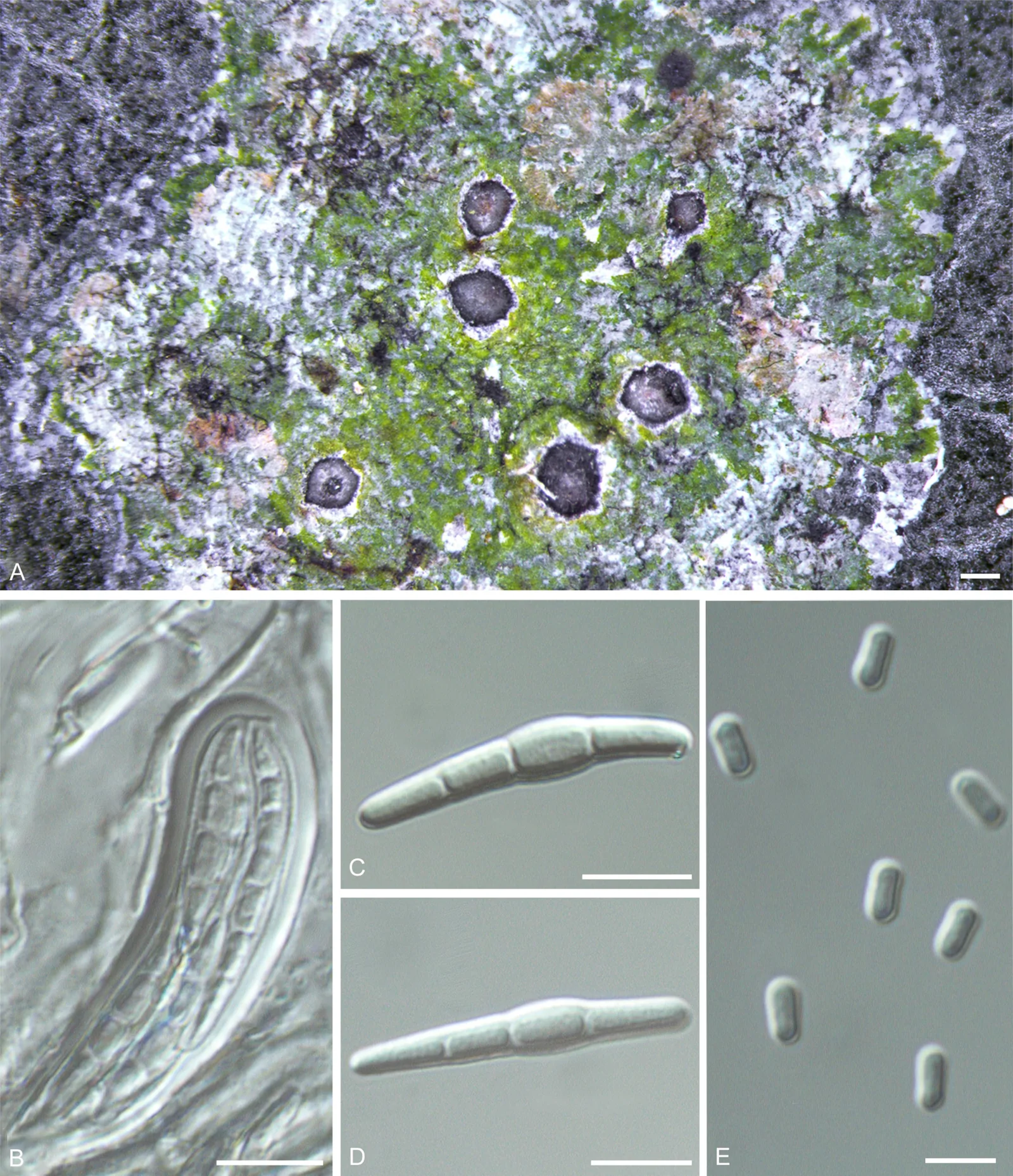
*Mazosia pruinata* sp. nov. (HMAS–L 152255). **A** Thallus with ascomata. **B** Ascus and ascospores. **C, D** Ascospores. **E** Macroconidia. Scale bars: 0.2 mm (**A**); 10 μm (**B–D**); 5 μm (**E**).

##### Chemistry.

No substances detected by TLC.

##### Habitat and distribution.

On unidentified angiosperm leaves in tropics; known only from the type locality, Hainan.

##### Additional material examined.

CHINA • Hainan Province, Ledong County, Jianfengling, alt. ca. 654 m, on living leaves, 10 Dec. 2019, Z.T. Yao HN20192668-2 (HMAS–L 152238), HN20192670-2 (HMAS–L 152239); alt. ca. 692 m, on living leaves, 10 Dec. 2019, Z.T. Yao HN20192701-1 (HMAS–L 152246); alt. ca. 783 m, on living leaves, 10 Dec. 2019, Z.T. Yao HN20192719-1 (HMAS–L 152253).

##### Notes.

Species similar to this new taxon, also with white pruina on the thallus, include *Mazosia papillosa*, *M. pseudotenuissima*, *M. pseudopruinata*, and *M. dispersa*. *Mazosia papillosa* has more prominent apothecia, larger asci (38–75 × 10–18 µm), and larger macroconidia (5–6 × 2.2–2.7 µm; see above); *M. pseudotenuissima* also has more prominent apothecia and larger asci (55–75 × 11–15 µm; see below); *M. pseudopruinata* differs in its thinner thallus (12.5–17.5 µm), more prominent apothecia (95–115 µm), and narrower asci (55–75 × 8.5–12 µm; see below); finally, *M. dispersa* is distinguished by larger verrucae (0.07–0.13 mm diam.) and often 5-septate ascospores (Lücking 2008).

#### Mazosia pseudoaptrootii

Taxon classificationAnimaliaArthonialesRoccellaceae

S.H. Jiang, Z.T. Yao, J.C. Wei & Lücking sp. nov.

13A3B2BD-8D24-5B06-BFBC-4EBF2EFABA1A

852574

Fig. 8

##### Etymology.

The epithet refers to the similarity with *Mazosia aptrootii*.

##### Typus.

CHINA • Hainan Province, Ledong County, Jianfengling, alt. ca. 960 m, on living leaves, 6 Sep. 2017, S.H. Jiang HN20171025-1 (holotype HMAS–L 152117).

##### Diagnosis.

The new species is similar to *Mazosia aptrootii* in its brown verrucae, but differs in its slightly narrower (18–25 × 3.8–5 µm vs. 20–27 × 5–6 µm), 3-septate ascospores.

##### Description.

Thallus foliicolous, continuous, pale yellowish brown, irregular rounded, 2–11 mm across, thin (15–23 µm), with dark brown prothallus, sometimes not developed; with brown verrucae (0.02–0.03 mm) and pilose; hairs formed by single, unbranched hyphae, colorless. Photobiont *Phycopeltis* sp., cells rectangular, 5–10 × 3–5 µm, in radiate plates. Ascomata rounded, 0.1–0.5 mm diam., and 90–100 µm high; disc dark grey, translucent when moistened; margin gently sloping outwards. Excipulum dark brown, 12.5–30 µm thick. Hypothecium pale brown, 12.5–22.5 μm high. Hymenium colorless, 70–90 µm high. Asci clavate, 55–70 × 10–15 µm, 8-spored. Ascospores fusiform, 3-septate, 18–25 × 3.8–5 µm, the second cell is slightly enlarged. Pycnidia low conical, 0.15–0.25 mm diam. Macroconidia bacillar, non-septate, 3.5–6 × 1.5–2.5 µm; Microconidia not seen.

**Figure 8.**
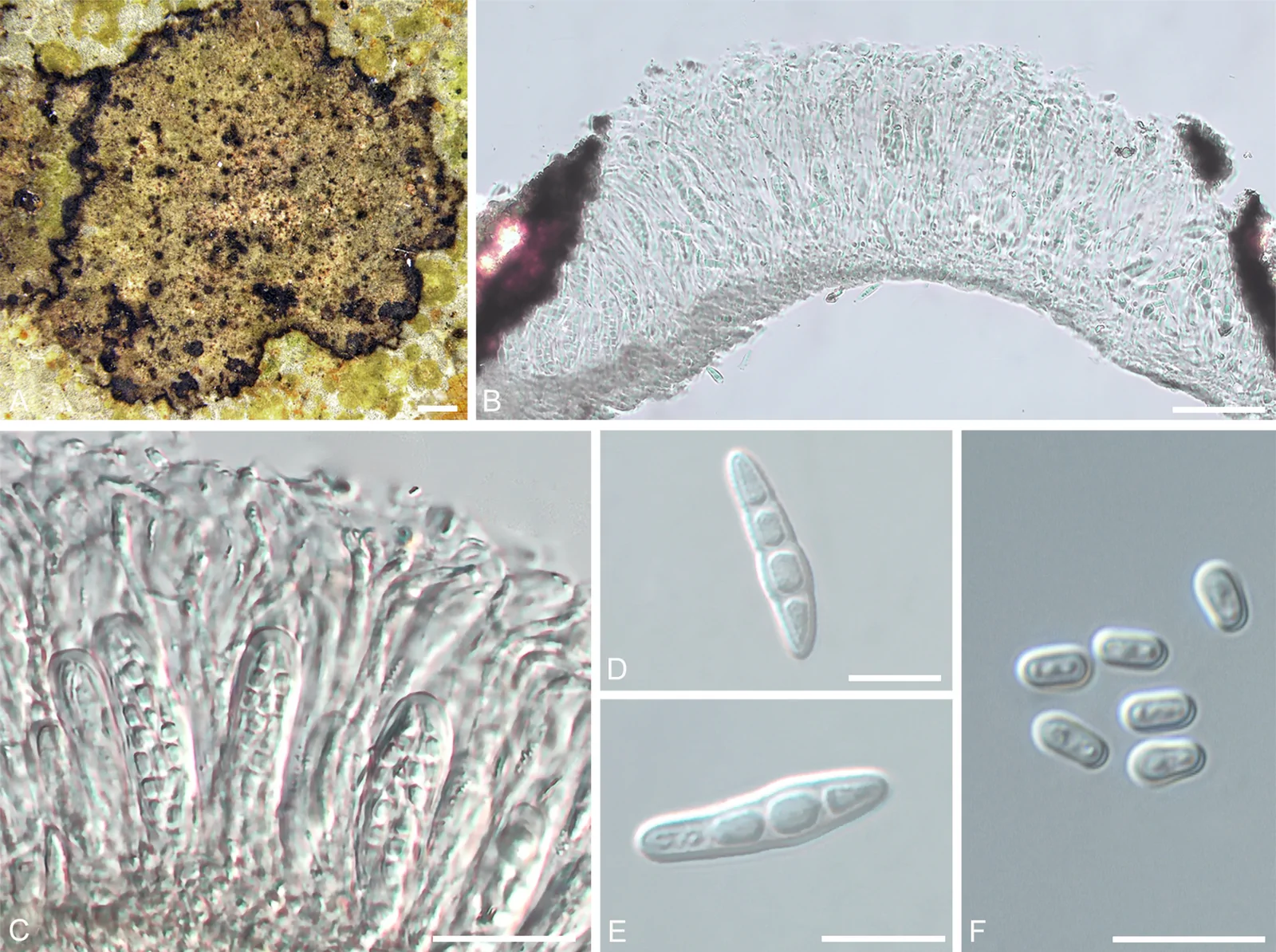
*Mazosia pseudoaptrootii* sp. nov. (HMAS–L 152117). **A** Thallus with ascomata. **B** Apothecium section. **C** Ascus and ascospores. **D, E** Ascospores. **F** Macroconidia. Scale bars: 0.5 mm (**A**); 50 μm (**B, C**); 10 μm (**D–F**).

##### Chemistry.

No substances detected by TLC.

##### Habitat and distribution.

On unidentified angiosperm leaves in (sub-)tropical regions of China.

##### Additional material examined.

CHINA • Hainan Province, Lingshui County, Diaoluo Mountain National Nature Reserve, alt. ca. 930 m, on living leaves, 31 Oct. 2016, X.Y Liu HN2016001-1 (HMAS–L 152202); alt. ca. 928 m, on living leaves, 14 Dec. 2019, Z.T. Yao HN20192875-1 (HMAS–L 152302), HN20192877-1 (HMAS–L 152303). Qiongzhong County, Limu Mountain National Nature Reserve, alt. ca. 800 m, on living leaves, 10 Sep. 2017, S.H. Jiang HN20170610-2 (HMAS–L 152051), HN20170625-2 (HMAS–L 152052), HN20170672-2 (HMAS–L 153486). Ledong County, Jianfengling, alt. ca. 960 m, on living leaves, 6 Sep. 2017, S.H. Jiang HN20171021-1 (HMAS–L 152116), HN20171032-2 (HMAS–L 152119), HN20171049-2 (HMAS–L 152062), HN20171059-3 (HMAS–L 152065), HN20171118-2 (HMAS–L 152074); alt. ca. 953 m, on living leaves, 9 Dec. 2019, Z.T. Yao HN20192603-1 (HMAS–L 153580), HN20192604-1 (HMAS–L 153558), HN20192608-2 (HMAS–L 153581), HN20192610-1 (HMAS–L 153582), HN20192611-1 (HMAS–L 152215), HN20192618-1 (HMAS–L 152218), HN20192620-1 (HMAS–L 153584), HN20192621-3 (HMAS–L 152219), HN20192622-1 (HMAS–L 153585), HN20192623-1 (HMAS–L 153586); alt. ca. 1019 m, on living leaves, 9 Dec. 2019, Z.T. Yao HN20192632-2 (HMAS–L 152225), HN20192636-1 (HMAS–L 152227), HN20192638-1 (HMAS–L 152228), HN20192642-1 (HMAS–L 152230), HN20192648-2 (HMAS–L 153588); alt. ca. 1049 m, on living leaves, 9 Dec. 2019, Z.T. Yao HN20192653-1 (HMAS–L 152233); alt. ca. 654 m, on living leaves, 10 Dec. 2019, Z.T. Yao HN20192682 (HMAS–L 152241), HN20192692-1 (HMAS–L 153592). Wuzhishan City, Wuzhi Mountain, alt. ca. 743 m, on living leaves, 12 Dec. 2019, Z.T. Yao HN20192740-1 (HMAS–L 152263), HN20192747-2 (HMAS–L 152265); alt. ca. 784 m, on living leaves, 12 Dec. 2019, Z.T. Yao HN20192775-1 (HMAS–L 152281), HN20192781-2 (HMAS–L 156348). Baoting County, Qixianling National Nature Reserve, alt. ca. 471 m, on living leaves, 13 Dec. 2019, Z.T. Yao HN20192860-1 (HMAS–L 153608). Guangxi Province, Shangsi County, Shiwan Mountain National Nature Reserve, alt. ca. 290 m, on living leaves, 21 Mar. 2019, S.H. Jiang & C. Zhang 20191078 (HMAS–L 156364).

##### Notes.

The main features of this new species are the pale yellowish-brown thallus with a dark brown prothallus, brown verrucae, a pilose surface, and 3-septate ascospores, 17.5–25 × 3.75–5 µm in size. Among the species with brown verrucae, it is most similar to *M. aptrootii*; however, the latter has slightly wider, (3–)5-septate ascospores (20–27 × 5–6 µm; Sipman 1991). Other taxa with brown verrucae are *M. bambusae*, *M. lueckingii*, and *M. quadriseptata*. While *M. bambusae* has a glabrous, thin thallus (7–15 µm; Lücking 2008), *M. lueckingii* differs by the larger (34–45 × 4–7 μm), (3–)5-septate ascospores (Singh and Pinokiyo 2008), and *M. quadriseptata* can be distinguished by the absence of a (dark) prothallus and 4-septate, larger ascospores (26–33 × 4–4.5 µm; Singh and Pinokiyo 2008). *Mazosia pseudobambusae* also has a dark prothallus, but its verrucae have the same color as the thallus (Lücking 2008).

#### Mazosia pseudocorticola

Taxon classificationAnimaliaArthonialesRoccellaceae

S.H. Jiang, Z.T. Yao, J.C. Wei & Lücking sp. nov.

34D6DCC1-8360-5B4E-88E7-170B776275DC

852575

Fig. 9

##### Etymology.

The epithet refers to the similarity with *Mazosia corticola*.

##### Typus.

CHINA • Hainan Province, Wuzhishan City, Wuzhi Mountain, alt. ca. 784 m, on living leaves, 12 Dec. 2019, C. Zhang & Z.T. Yao 20192319 (holotype HMAS–L 153577).

##### Diagnosis.

*Mazosia pseudocorticola* is similar to *M. corticola* regarding the corticolous habitat ecology and the asci, but can be distinguished by the 3–5-septate ascospores, and unknown substance detected by TLC.

##### Description.

Thallus crustose, corticolous, green to gray green, distinct cortex, with irregular cracks, coarse verrucae (0.05–0.12 mm). Photobiont *Trentepohlia* sp. Ascomata rounded, 0.08–0.5 mm diam.; disc black, white at the margin. Excipulum brown, 13–20 µm thick. Hypothecium pale brown, 10–30 μm high. Hymenium colorless, 70–100 µm high, K–, I+ orange-red, KI+ orange-yellow and part blue. Paraphysis branched. Asci clavate, 50–75 × 12–17 µm, 8-spored. Ascospores fusiform, 3–5-septate, 20–28 × 5–6.5 µm, the second cell is slightly enlarged. Pycnidia not seen.

**Figure 9.**
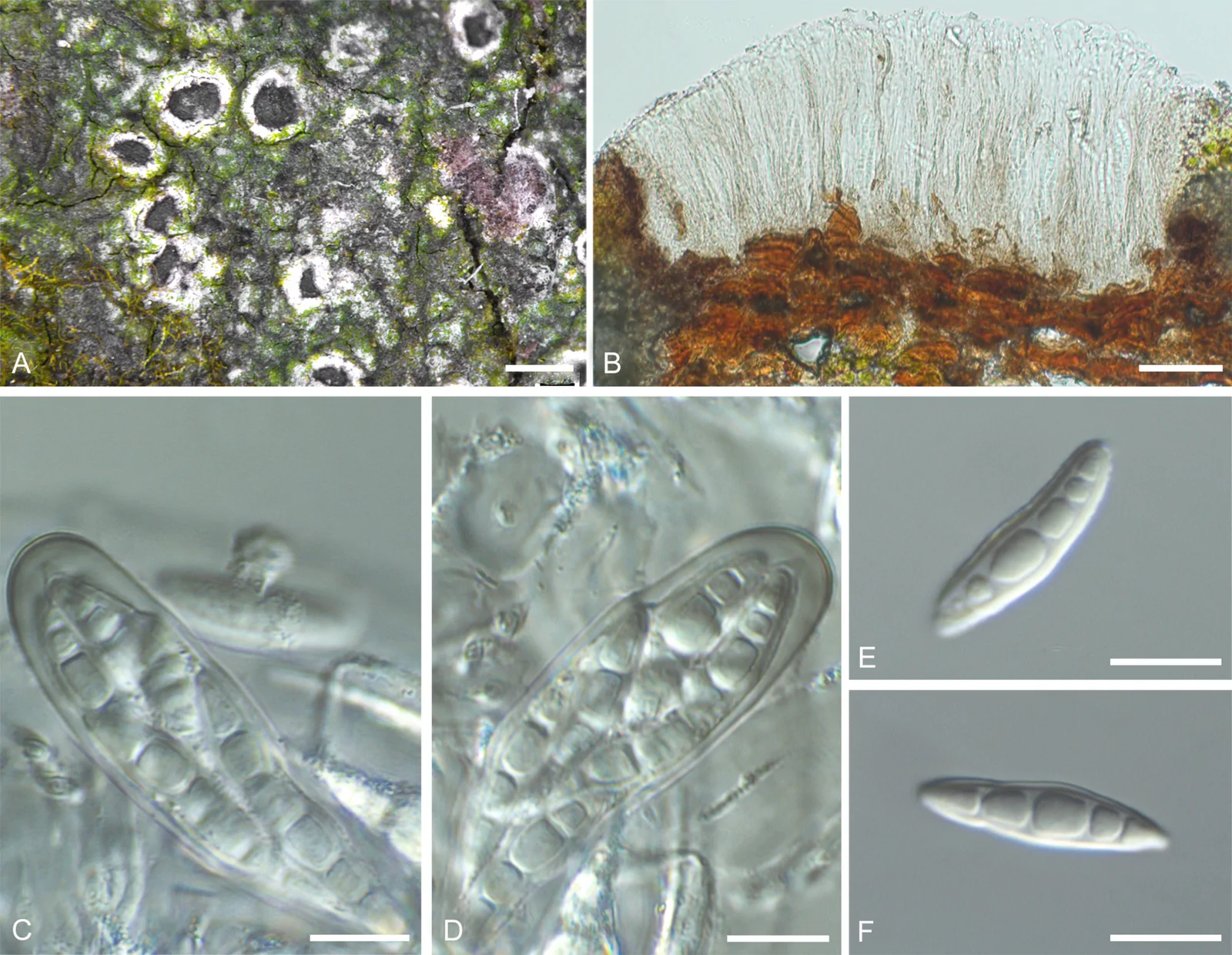
*Mazosia pseudocorticola* sp. nov. (HMAS–L 153577). **A** Thallus with ascomata. **B** Apothecium section. **C, D** Ascus. **E, F** Ascospores. Scale bars: 0.4 mm (**A**); 50 μm (**B**); 10 μm (**C–F**).

##### Chemistry.

Thallus K+ brown, C–; disc white margin P+ yellow; medulla P+ yellow. An unknown substance detected in region 5–6 in solvent C by TLC.

##### Habitat and distribution.

On the bark in tropics; known only from the type locality, Hainan.

##### Notes.

There are nine *Mazosia* species known to occur on bark, but only *M. ocellata* was reported in China before (Aptroot and Sparrius 2003; Aptroot et al. 2014). The latter differs from this new species by its 3-septate, smaller ascospores (17–21 × 4–4.5 μm), and no substances are detectable in TLC (Aptroot et al. 2014). *Mazosia corticola* agrees with the new species in the corticolous habitat ecology and asci, but can be recognized by the 3-septate ascospores, and psoromic acid detected by TLC (Kantvilas 2020). Other similar corticolous species are *Mazosia bruguierae*, *M. carnea*, and *M. japonica*, but all have smaller ascospores and lack lichen substances (Aptroot et al. 2014; Sakata et al. 2017).

#### Mazosia pseudomelanophthalma

Taxon classificationAnimaliaArthonialesRoccellaceae

S.H. Jiang, Z.T. Yao, J.C. Wei & Lücking sp. nov.

292887AC-20FA-5C97-A168-4ACE62185B48

852576

Fig. 10

##### Etymology.

The epithet refers to the similarity with *Mazosia melanophthalma*.

##### Typus.

CHINA • Hainan Province, Baoting County, Qixianling National Nature Reserve, alt. ca. 303 m, on living leaves, 13 Dec. 2019, Z.T. Yao HN20192805-1 (holotype HMAS–L 152284).

##### Diagnosis.

The new species is similar to *Mazosia melanophthalma*, but differs in the smaller thallus (1.5–7 mm vs. 5–15 mm), higher ascomata (100–115 µm vs. 60–90 µm), and narrower macroconidia (5–7 × 1.5–2 µm vs. 5–8 × 2–3 µm).

##### Description.

Thallus foliicolous, continuous, yellowish green, rounded, the color of the margin paler than the center, 1.5–7 mm across, thin (12.5–17.5 µm); verrucae 0.05–0.1 mm diam., with the same color of thallus, those at the margin are slightly elongated and arranged radially. Photobiont *Phycopeltis* sp., cells rectangular, 10–15 × 3.8–5 µm, in radiate plates. Ascomata rounded, 0.07–0.25 mm diam., and 100–115 µm high; disc dark grey, translucent when moistened; margin gently sloping outwards. Excipulum dark brown, 17–22 µm thick. Hypothecium pale brown, 11–18 µm high. Hymenium colorless, 75–95 µm high. Asci clavate, 45–53 × 12.5–15 µm, 8-spored. Ascospores fusiform, 3-septate, 14–22 × 3.8–5 µm. Pycnidia low conical, 0.05–0.1 mm diam. Macroconidia bacillar, non-septate, 5–7 × 1.5–2 µm; Microconidia 2.5–3 × 1–1.5 µm. Macroconidia and microconidia often appear in the same pycnidia.

**Figure 10.**
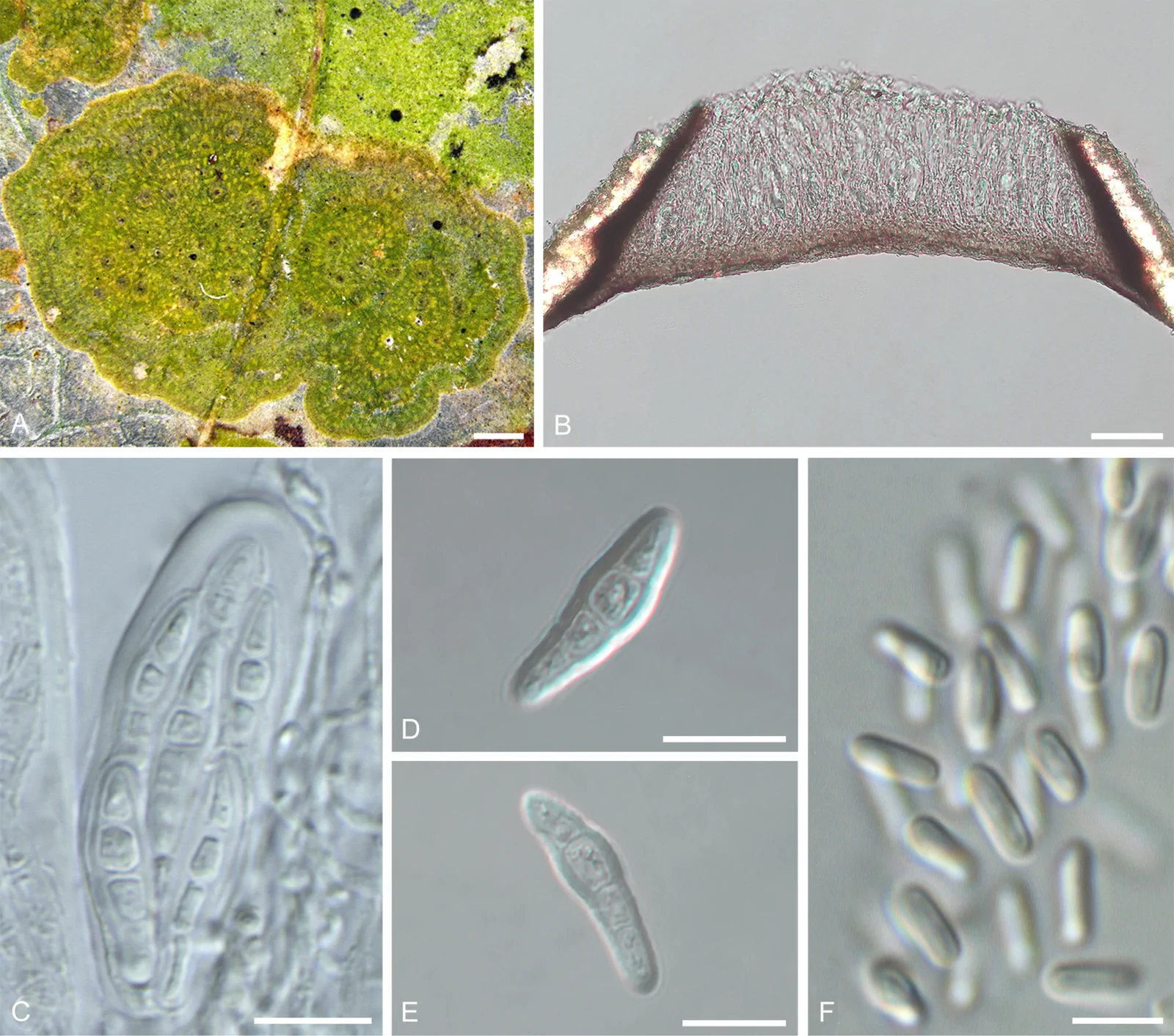
*Mazosia pseudomelanophthalma* sp. nov. (HMAS–L 152284). **A** Thallus with ascomata. **B** Apothecium section. **C** Ascus. **D, E** Ascospores. **F** Macroconidia. Scale bars: 0.5 mm (**A**); 50 μm (**B**); 10 μm (**C–E**); 5 μm (**F**).

##### Chemistry.

No substances detected by TLC.

##### Habitat and distribution.

On unidentified angiosperm leaves in tropics; known only from the type locality, Hainan.

##### Additional material examined.

CHINA • Hainan Province, Baoting County, Qixianling National Nature Reserve, alt. ca. 350 m, on living leaves, 7 Sep. 2017, S.H. Jiang HN20171293 (HMAS–L 152080), HN20171494-1 (HMAS–L 152092), HN20171518 (HMAS–L 152144), HN20171562-2 (HMAS–L 152147), HN20171620 (HMAS–L 152093); alt. ca. 303 m, on living leaves, 13 Dec. 2019, Z.T. Yao HN20192797-1 (HMAS–L 156349), HN20192805-2 (HMAS–L 156350), HN20192806-1 (HMAS–L 152285). Wuzhishan City, Wuzhi Mountain, alt. ca. 743 m, on living leaves, 12 Dec. 2019, Z.T. Yao HN20192741-2 (HMAS–L 153600).

##### Notes.

This new species is very similar to *Mazosia melanophthalma*, but both form distinct linages in the molecular phylogeny. The latter often has a larger thallus (5–15 mm), lower ascomata (60–90 µm), and wider macroconidia (5–8 × 2–3 µm; Henssen and Jahns 1974). *Mazosia dispersa* is also related to the new species, but can be distinguished by its thallus often being pruinose and by its larger ascospores (26–39 × 4–7 µm; Lücking 2008). Another similar species is *M. pseudopapillosa*, but that taxon differs in having longer ascospores (23–30 × 2.5–5 µm; see below). *Mazosia hainanensis* is also similar to the new species, but has smaller verrucae (0.02–0.03 mm) and smaller ascospores (14–18.5 × 3–4 µm; Yao et al. 2021b). *Mazosia gelatinospora* chiefly differs from the new species in its ascospores being surrounded by a gelatinous sheath (see above). The new species is also similar to *M. verrucosa*, but the latter has smaller thallus (0.3–2 mm), smaller verrucae (0.03–0.05 mm), and narrower asci (40–53 × 9.5–11.5 µm; see below).

#### Mazosia pseudopapillosa

Taxon classificationAnimaliaArthonialesRoccellaceae

S.H. Jiang, Z.T. Yao, J.C. Wei & Lücking sp. nov.

5FC59D79-27C1-5D10-A471-9D14A2D084F6

852577

Fig. 11

##### Etymology.

The epithet refers to the similarity with *Mazosia papillosa*.

##### Typus.

CHINA • Hainan Province, Wuzhishan City, Wuzhi Mountain, alt. ca. 600 m, on living leaves, 9 Sep. 2017, S.H. Jiang HN20170692-1 (holotype HMAS–L 146148).

##### Diagnosis.

The new species is similar to *Mazosia papillosa*, but can be distinguished by verrucose thallus lacks pilose.

##### Description.

Thallus foliicolous, yellow-green or brown-yellow mottled with white, without prothallus, but there is a brown-black border with other lichens, 3–5 mm across, finely verrucose; verrucae 0.04–0.07 mm, slightly paler than the thallus. Photobiont *Phycopeltis* sp., cells rectangular, 7.5–18 × 5–7.5 µm, in radiate plates. Ascomata rounded, 0.17–0.22 mm diam., and 90–100 µm high; disc dark grey to black, margin slightly higher than disc and gently sloping outwards. Excipulum dark brown, 12.5–25 µm thick. Hypothecium colorless to brown, 15–25 µm high. Hymenium colorless, 65–75 µm high. Asci clavate, 45–55 × 11–14 µm, 8-spored. Ascospores fusiform, 3-septate, 23–30 × 2.5–5 µm, the second cell is slightly enlarged. Pycnidia low conical, 0.2–0.4 mm diam. Macroconidia bacillar, non-septate, 3.8–5 × 2–2.5 µm. Microconidia not seen.

**Figure 11.**
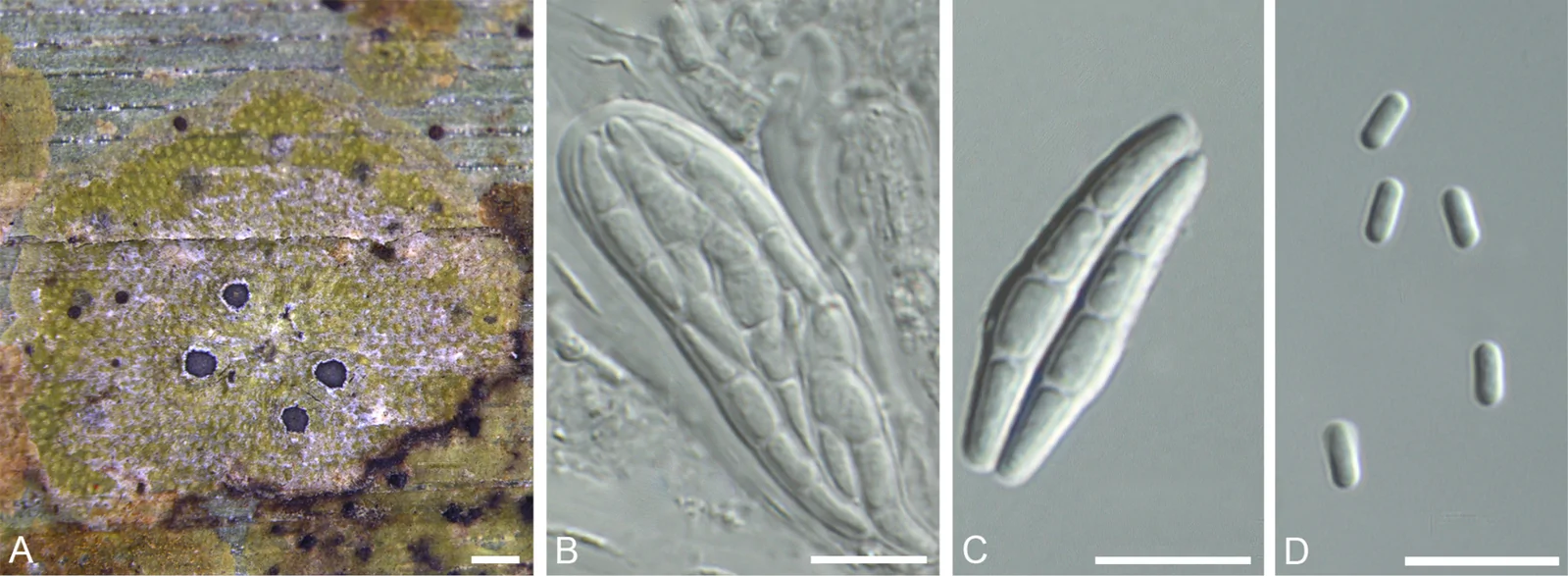
*Mazosia pseudopapillosa* sp. nov. (HMAS–L 146148). **A** Thallus with ascomata. **B** Ascus. **C** Ascospores. **D** Macroconidia. Scale bars: 0.2 mm (**A**); 10 μm (**B–D**).

##### Chemistry.

No substances are detected by TLC.

##### Habitat and distribution.

On unidentified angiosperm leaves in (sub-)tropical regions of China.

##### Additional material examined.

CHINA • Hainan Province, Wuzhishan City, Wuzhi Mountain, alt. ca. 600 m, on living leaves, 9 Sep. 2017, S.H. Jiang HN20170691-1 (HMAS–L 146149). Yunnan Province, Honghezhou, Daweishan, Shazhudi, alt. ca. 890 m, on living leaves, 23 Sep. 2011, Z.F. Jia 11-470 (LCUF).

##### Notes.

The species is similar to *Mazosia papillosa*, but the latter often has fine hairs formed by fungal hyphae in addition to verrucae (see above). *Mazosia melanophthalma* also resembles the new species in morphology, but it has larger verrucae (0.05–0.1 mm) and smaller ascospores (15–22 × 3–5 µm; Lücking 2008). *Mazosia pseudobambusae* differs in its pale brownish gray thallus, dark brown prothallus, and the slightly wider ascospores (20–28 × 4–5 µm; Lücking 2008).

#### Mazosia pseudopilosa

Taxon classificationAnimaliaArthonialesRoccellaceae

S.H. Jiang, Z.T. Yao, J.C. Wei & Lücking sp. nov.

6A0EA42A-4E20-5430-A855-DFE381AD97EC

852578

Fig. 12

##### Etymology.

The epithet refers to the similarity with *Mazosia pilosa*.

##### Typus.

CHINA • Hainan Province, Wuzhishan City, Wuzhi Mountain, alt. ca. 743 m, on living leaves, 12 Sep. 2019, Z.T. Yao HN20192751-1 (holotype HMAS–L 152267).

##### Diagnosis.

The new species externally resembles *Mazosia pilosa* in a pilose thallus without verrucae, but differs by the 3–7-septate, longer ascospores (24–50 × 4.5–6 µm vs. 17–27 × 3.5–5 µm), and an unidentified substance detected by TLC.

##### Description.

Thallus crustose, foliicolous, pale yellow, rounded, smaller thallus often aggregate to form larger patches, and surrounded by scattered smaller thallus, 0.05–4 mm across, 10–18 µm thick, without verrucae, pilose (sometimes not distinct); hairs formed by single, unbranched hyphae. Photobiont *Phycopeltis* sp., cells rectangular, 10–15 × 2.5–5 µm, in radiate plates. Ascomata rounded, 0.05–0.15 mm diam., and 85–120 µm high; disc black, translucent when moistened and gently sloping outwards. Excipulum dark brown, 7.5–15 µm thick. Hypothecium pale brown, 7.5–12.5 µm high. Hymenium colorless, 85–100 µm high. Asci clavate, 55–80 × 15–18 µm, 8-spored. Ascospores fusiform, 3–7-septate, 24–50 × 4.5–6 µm, the second cell is slightly enlarged. Pycnidia not seen.

**Figure 12.**
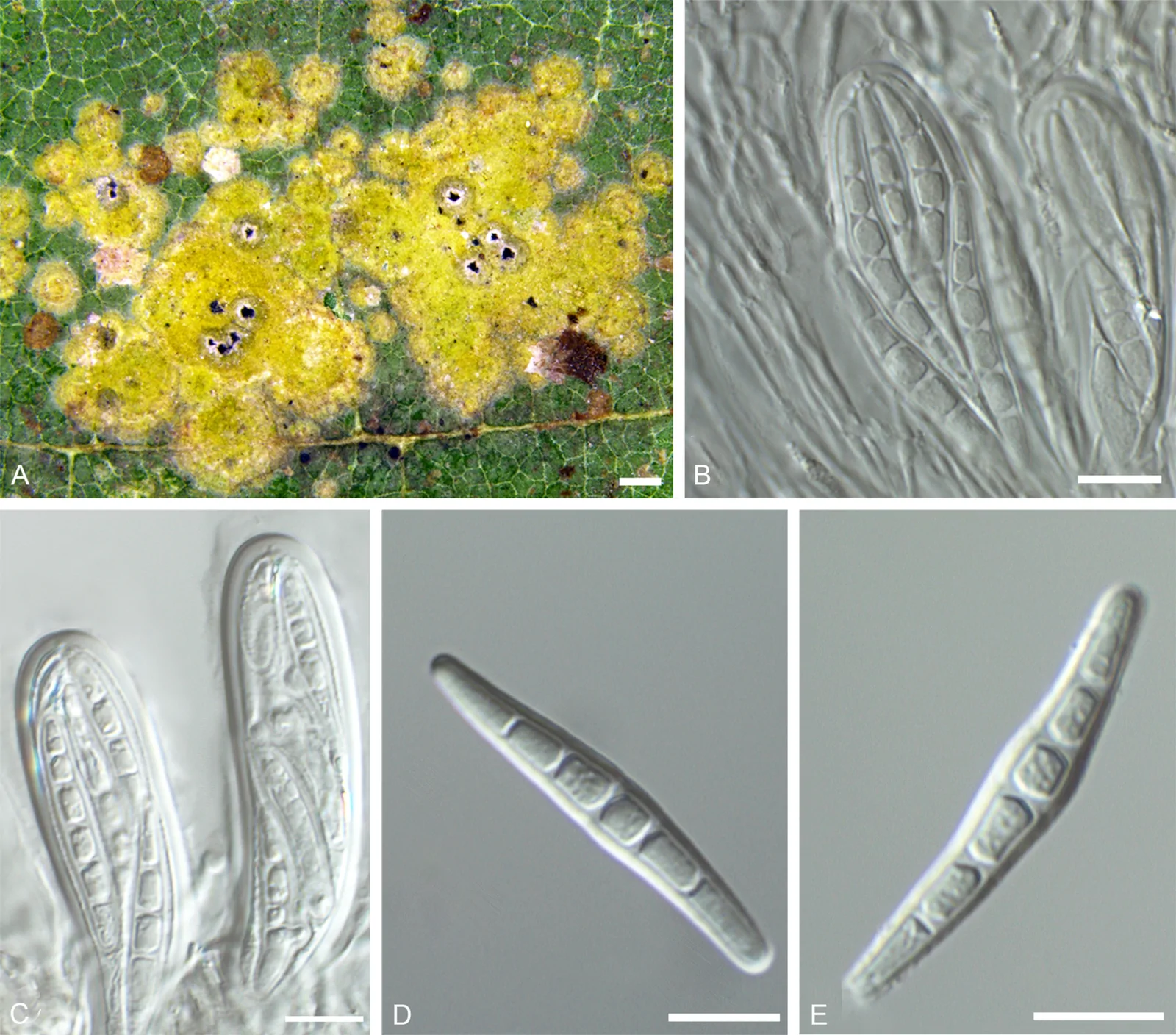
*Mazosia pseudopilosa* sp. nov. (HMAS–L 152267). **A** Thallus with ascomata. **B** Apothecium section. **C** Ascus and ascospores. **D, E** Ascospores. Scale bars: 0.4 mm (**A**); 10 μm (**B–E**).

##### Chemistry.

An unidentified substance detected by TLC.

##### Habitat and distribution.

On unidentified angiosperm leaves in tropics; known only from the type locality, Hainan.

##### Additional material examined.

CHINA • Hainan Province, Wuzhishan City, Wuzhi Mountain, alt. ca. 743 m, on living leaves, 12 Sep. 2019, Z.T. Yao HN20192742-1 (HMAS–L 152264). Ledong County, Jianfengling, alt. ca. 960 m, on living leaves, 6 Sep. 2017, S.H. Jiang HN20171085 (HMAS–L 152073).

##### Notes.

*Mazosia pseudopilosa* strongly resembles *M. pilosa* in a pilose thallus without verrucae, but the latter can be distinguished by its 3-septate, shorter ascospores (17–27 × 3.5–5 µm), and no substances detected by TLC (Lücking 2008). There are five other species in *Mazosia* that agree with this new species in having a pilose thallus: *Mazosia aptrootii*, *M. tenuissima*, *M. tomentifera*, *M. uniseptata* and *M. weii*. *Mazosia aptrootii* has brown verrucae and 3–5-septate, shorter ascospores (20–27 × 5–6 µm; Sipman 1991); *M. tenuissima* is charactized by a verrucose thallus and 3-septate, shorter ascosores (18–26 × 3.5–5 µm; Lücking 2008); *M. tomentifera* differs in its 5-septate ascospores (Lumbsch and Vězda 1990; Lücking 2008); *M. uniseptata* typically has 1-septate and shorter ascospores (Lücking 2008); and *M. weii* differs in its thallus with brown verrucae and shorter ascospores (22.5–35 × 4–6.25 µm; Yao et al. 2021a).

#### Mazosia pseudopruinata

Taxon classificationAnimaliaArthonialesRoccellaceae

S.H. Jiang, Z.T. Yao, J.C. Wei & Lücking sp. nov.

EDFCC7AF-1CDB-5B8E-944D-BA0F7368F048

852579

Fig. 13

##### Etymology.

The epithet refers to the similarity with *Mazosia pruinata*.

##### Typus.

CHINA • Hainan Province, Ledong County, Jianfengling, alt. ca. 783 m, on living leaves, 10 Dec. 2019, Z.T. Yao HN20192724-1 (holotype HMAS–L 152256).

##### Diagnosis.

The species is similar to *Mazosia pruinata*, but can be distinguished by the larger ascomata (0.15–0.5 mm × 95–115 µm vs. 0.1–0.22 × 80–90 µm), higher hymenium (90–100 µm vs. 60–70 µm), longer asci (55–75 µm vs. 39–58 µm), and 3-septate, occasionally 1–2-septate ascospores.

##### Description.

Thallus crustose, foliicolous, pale yellow-green, rounded, 3–5 mm across, 12.5–17.5 µm thick, with white pruina, verrucae, pilose (sometimes not distinct); verrucae 0.04–0.07 mm, the top is white or with the same color of thallus. Photobiont *Phycopeltis* sp., cells rectangular, 10–15 × 5–7.5 µm, in radiate plates. Ascomata rounded, 0.15–0.5 mm diam., and 95–115 µm high; disc dark grey, translucent when moistened and gently sloping outwards. Excipulum dark brown, 10–15 µm thick. Hypothecium pale brown, 12–20 µm high. Hymenium colorless, 90–100 µm high. Asci clavate, 55–75 × 8.5–12 µm, 8-spored. Ascospores fusiform, 3-septate, occasionally 1–2-septate, 19–38 × 3–5 µm, the second cell is slightly enlarged. Pycnidia low conical, 0.03–0.05 mm diam. Macroconidia bacillar, non-septate, 4–5.5 × 2–2.5 µm. Microconidia fusiform-ellipsoid, non-septate, 2.5–3 × 1–1.5 µm.

**Figure 13.**
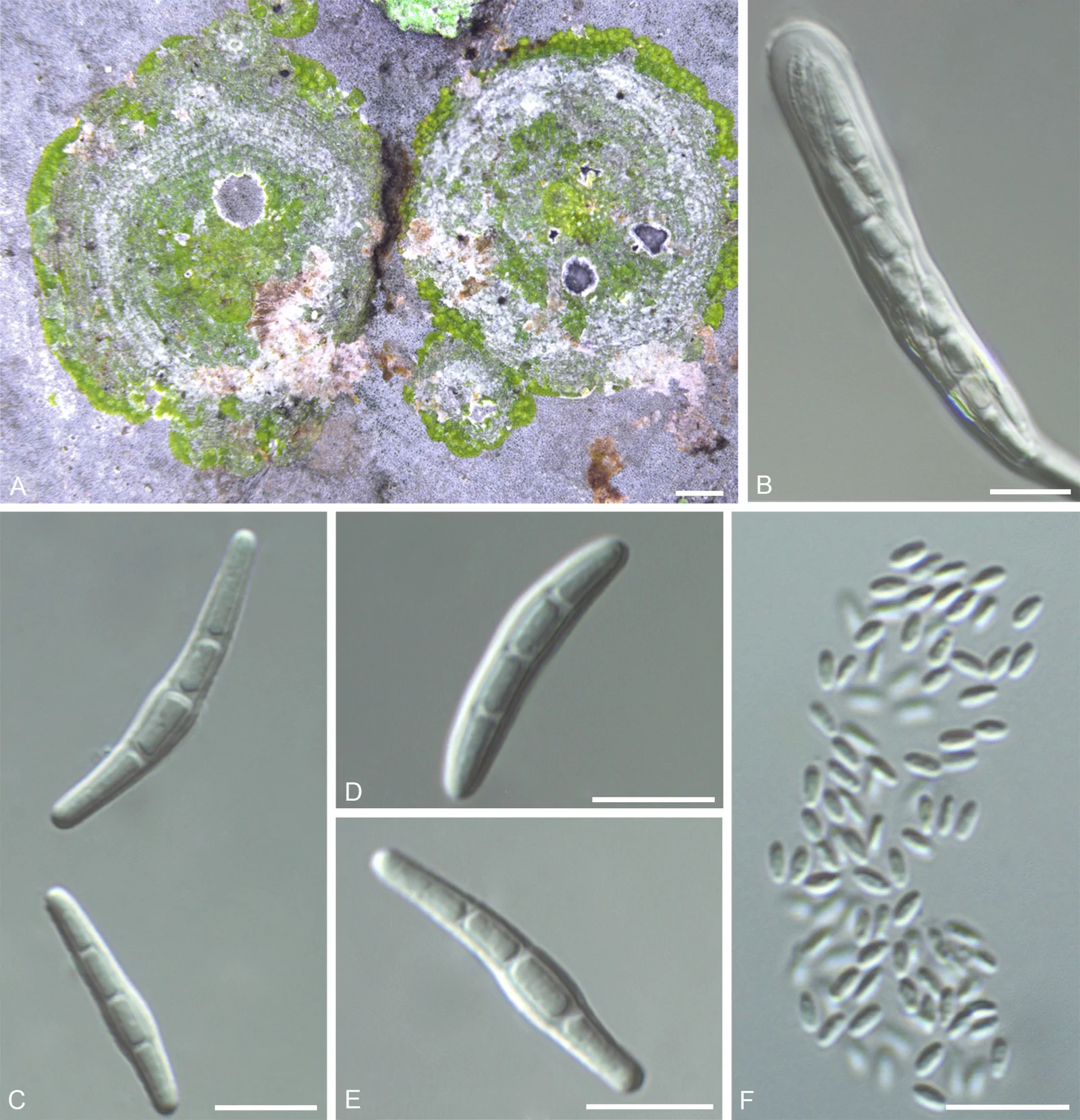
*Mazosia pseudopruinata* sp. nov. (HMAS–L 152256). **A** Thallus with ascomata. **B** Ascus. **C–E** Ascospore. **F** Microconidia. Scale bars: 0.4 mm (**A**); 10 μm (**B–F**).

##### Chemistry.

No substances detected by TLC.

##### Habitat and distribution.

On unidentified angiosperm leaves in (sub-)tropical regions of China.

##### Additional material examined.

CHINA • Hainan Province, Ledong County, Jianfengling, alt. ca. 692 m, on living leaves, 10 Dec. 2019, Z.T. Yao HN20192698-1 (HMAS–L 152244), HN20192700-1 (HMAS–L 152245); alt. ca. 783 m, on living leaves, 10 Dec. 2019, Z.T. Yao HN20192713-1 (HMAS–L 152250), HN20192714-2 (HMAS–L 152251), HN20192726-1 (HMAS–L 152257), HN20192728-1 (HMAS–L 153597), HN20192729-1 (HMAS–L 152258). Baoting County, Qixianling National Nature Reserve, alt. ca. 471 m, on living leaves, 13 Dec. 2019, Z.T. Yao HN20192842-6 (HMAS–L 153532), HN20192849-3 (HMAS–L 153609), HN20192850-1 (HMAS–L 153574), HN20192861-2 (HMAS–L 153611). Lingshui County, Diaoluo Mountain National Nature Reserve, alt. ca. 928 m, on living leaves, 14 Dec. 2019, Z.T. Yao HN20192878-1 (HMAS–L 152304). Wuzhishan City, Wuzhi Mountain, alt. ca. 743 m, on living leaves, 12 Sep. 2019, Z.T. Yao HN20192757-2 (HMAS–L 156346). Guangdong Province, Shixing County, Chebaling National Nature Reserve, alt. ca. 420 m, on living leaves, 19 Mar. 2019, S.H. Jiang & C. Zhang 20190926 (HMAS–L 156358), 20190949-1 (HMAS–L 156359).

##### Notes.

This new species is most similar to *Mazosia pruinata*, especially in its pruinose thallus, but the latter has smaller ascomata (0.1–0.22 mm diam. and 80–90 µm high), a lower hymenium (60–70 µm high), shorter asci (39–58 × 12–15 µm), and 3–(4–5)-septate ascospores (see above). *Mazosia dispersa* is another similar species in having a thallus with pruina, but can be distinguished by its (4–)5(–7)-septate ascospores (26–39 × 4–7 µm; Lücking 2008). The new species is also similar with *Mazosia tenuissima* in verrucae and pilose, but the latter has thinner thallus (5–10 µm) without pruina, lower ascomata, shorter ascospores (18–26 × 3.5–5 µm) and longer macroconidia (5–8 × 2–3 µm; Lücking 2008). Another similar species *Mazosia pilosa* differs in thallus without verrucae, and shorter ascospores (17–27 × 3.5–5 µm; Lücking 2008). *Mazosia melanophthalma* agrees with this new species in the verrucose thallus, but lacks hairs and has shorter asci (45–55 × 8–12 µm), shorter ascospores (15–22 × 3–5 µm), and longer macroconidia (5–8 × 2–3 µm; Lücking 2008). *Mazosia pseudobambusae* also has verrucae; however, the thallus margin is irregular, with a dark brown prothallus, and the ascospores are shorter (20–28 × 4–5 µm; Lücking 2008).

#### Mazosia pseudotenuissima

Taxon classificationAnimaliaArthonialesRoccellaceae

S.H. Jiang, Z.T. Yao, J.C. Wei & Lücking sp. nov.

9E185224-A319-5532-8F1D-9596AA68F475

852580

Fig. 14

##### Etymology.

The epithet refers to the similarity with *Mazosia tenuissima*.

##### Typus.

CHINA • Hainan Province, Ledong County, Jianfengling, alt. ca. 953 m, on living leaves, 9 Dec. 2019, Z.T. Yao HN20192600-1 (holotype HMAS–L 147861).

##### Diagnosis.

The new species is similar to *Mazosia tenuissima*, but can be distinguished by the thicker thallus (10–14 µm thick vs. 5–10 µm), higher ascomata (105–125 µm high vs. 60–80 µm), and longer asci (55–75 × 11–15 µm vs. 45–55 × 10–13 µm).

##### Description.

Thallus crustose, foliicolous, dark yellow-green, rounded, rough, white at the margin, 2.5–4 mm across, 10–14 µm thick, with verrucae, pilose (sometimes not distinct); verrucae 0.04–0.07 mm, white at the top in the central. Photobiont *Phycopeltis* sp., cells rectangular, 10–17.5 × 5–10 µm, in radiate plates. Ascomata rounded, 0.1–0.3 mm diam., and 105–125 µm high; disc dark grey, translucent when moistened and gently sloping outwards. Excipulum dark brown, 12–29 µm thick. Hypothecium pale brown, 15–21 µm high. Hymenium colorless, 90–100 µm high. Asci clavate, 55–75 × 11–15 µm, 8-spored. Ascospores fusiform, 3-septate, 22–30 × 4–5.5 µm, the second cell is slightly enlarged. Pycnidia low conical, 0.03–0.06 mm diam. Macroconidia bacillar, non-septate, 4–4.7 × 1.7–2.2 µm. Microconidia not seen.

**Figure 14.**
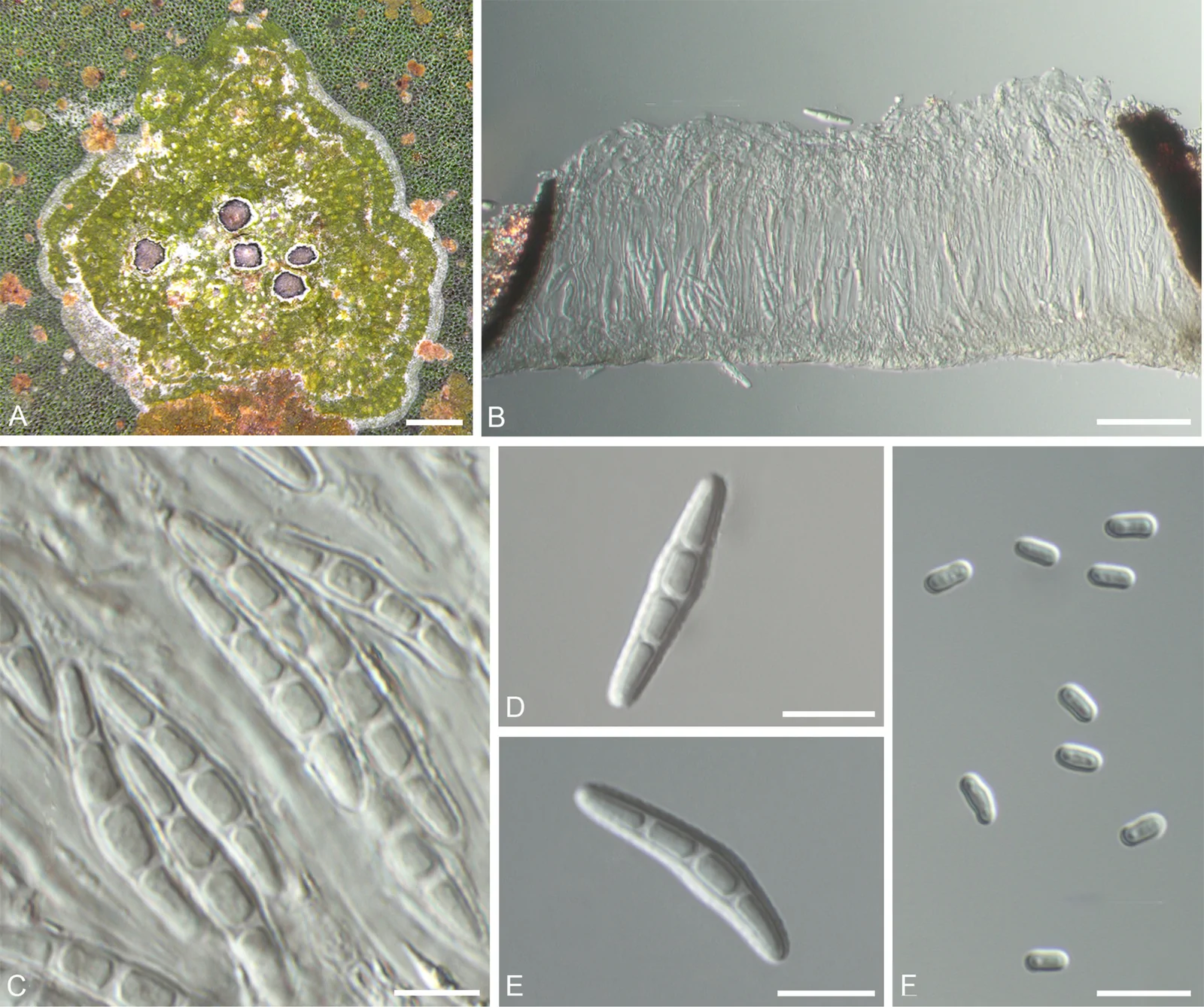
*Mazosia pseudotenuissima* sp. nov. (HMAS–L 147861). **A** Thallus with ascomata. **B** Apothecium section. **C** Asci and ascospores. **D, E** Ascospores. **F** Macroconidia. Scale bars: 0.5 mm (**A**); 50 μm (**B**); 10 μm (**C–F**).

##### Chemistry.

No substances detected by TLC.

##### Habitat and distribution.

On unidentified angiosperm leaves in (sub-)tropical regions of China.

##### Additional material examined.

CHINA • Hainan Province, Ledong County, Jianfengling, alt. ca. 953 m, on living leaves, 9 Dec. 2019, Z.T. Yao HN20192609-1 (HMAS–L 153559), HN20192613 (HMAS–L 153560), HN20192619-1 (HMAS–L 153561); alt. ca. 960 m, on living leaves, 6 Sep. 2017, S.H. Jiang HN20171090-1 (HMAS–L 153542); alt. ca. 1019 m, on living leaves, 9 Dec. 2019, Z.T. Yao HN20192635-1 (HMAS–L 153562), HN20192637-1 (HMAS–L 153508), HN20192639 (HMAS–L 153509), HN20192643-1 (HMAS–L 147863), HN20192645-1 (HMAS–L 153563); alt. ca. 1049 m, on living leaves, 9 Dec. 2019, Z.T. Yao HN20192650-2 (HMAS–L 147862); alt. ca. 654 m, on living leaves, 10 Dec. 2019, Z.T. Yao HN20192667-1 (HMAS–L 153511); alt. ca. 692 m, on living leaves, 10 Dec. 2019, Z.T. Yao HN20192710-3 (HMAS–L 147864). Wuzhishan City, Wuzhi Mountain, alt. ca. 784 m, on living leaves, 12 Dec. 2019, Z.T. Yao HN20192779-2 (HMAS–L 153564). Lingshui County, Diaoluo Mountain National Nature Reserve, alt. ca. 928 m, on living leaves, 14 Dec. 2019, Z.T. Yao HN20192868-1 (HMAS–L 153539). Yunnan Province, Xishuangbanna, Mengla County, Tropical Botanical Garden of Chinese Academy of Sciences, alt. ca. 689 m, on living leaves, 28 January 2018, X.H. Wu YN18284 (LCUF).

##### Notes.

This new species is similar to *Mazosia tenuissima*, but the latter differs in its thinner thallus (5–10 µm), the verrucae not becoming white at the top, its lower ascomata (60–80 µm), and shorter asci (45–55 × 10–13 µm; Lücking 2008). *Mazosia pseudotenuissima* also resembles *M. papillosa*, but the latter has pruinose thallus, smaller verrucae (0.02–0.05 mm in diam.), and broader macroconidia (5–6 × 2.2–2.7 µm; see above). *Mazosia aptrootii*, *M. pseudopilosa*, and *M. weii* are also similar to this new species in their pilose thalli, but *M. aptrootii* has brown verrucae and 3–5-septate ascospores (Sipman 1991), *M. pseudopilosa* lacks verrucae and has 3–7-septate, larger ascospores (24–50 × 4.5–6 µm; see above), and *M. weii* has brown verrucae and an unknown substance detected by TLC (Yao et al. 2021a).

#### Mazosia sinensis

Taxon classificationAnimaliaArthonialesRoccellaceae

S.H. Jiang, Z.T. Yao, J.C. Wei & Lücking sp. nov.

0C907565-8AAA-5AE8-9760-2A9CFF0D4861

852581

Fig. 15

##### Etymology.

The specific epithet is derived from the country of the type locality of the species, China.

##### Typus.

CHINA • Hainan Province, Wuzhishan City, Wuzhi Mountain, alt. ca. 890 m, on living leaves, 12 Dec. 2019, Z.T. Yao HN20192783-1 (holotype HMAS–L 152283).

##### Diagnosis.

This new species is similar to *Mazosia bambusae*, but can be distinguished by smaller thallus (2.5–5 mm vs. 5–20 µm) and smaller apothecia (0.08–0.2 mm vs. 0.3–0.6 mm).

##### Description.

Thallus crustose, foliicolous, pale yellowish-green, sometimes white at the margin, rounded, 2.5–5 mm across, 10–18 µm thick, with a black prothallus, verrucae, slightly pilose (sometimes indistinct); verrucae 0.01–0.04 mm, brown at the top in the centre. Photobiont *Phycopeltis* sp., cells rectangular, 10–15 × 3.8–5 µm, in radiate plates. Ascomata rounded, 0.08–0.2 mm diam., and 90–100 µm high; disc dark grey to black, translucent when moistened and gently sloping outwards. Excipulum dark brown, 11–21 µm thick. Hypothecium colorless, 7.5–12.5 µm high. Hymenium colorless, 70–85 µm high. Asci clavate, 45–50 × 10–12 µm, 8-spored. Ascospores fusiform, 3-septate, 15–23 × 3.8–5 µm, the second cell is slightly enlarged. Pycnidia low conical, 0.05–0.1 mm diam. Macroconidia bacillar, non-septate, 5–6.5 × 2–2.5 µm. Microconidia colorless, non-septate, 2–2.5 × 0.5–1 µm.

**Figure 15.**
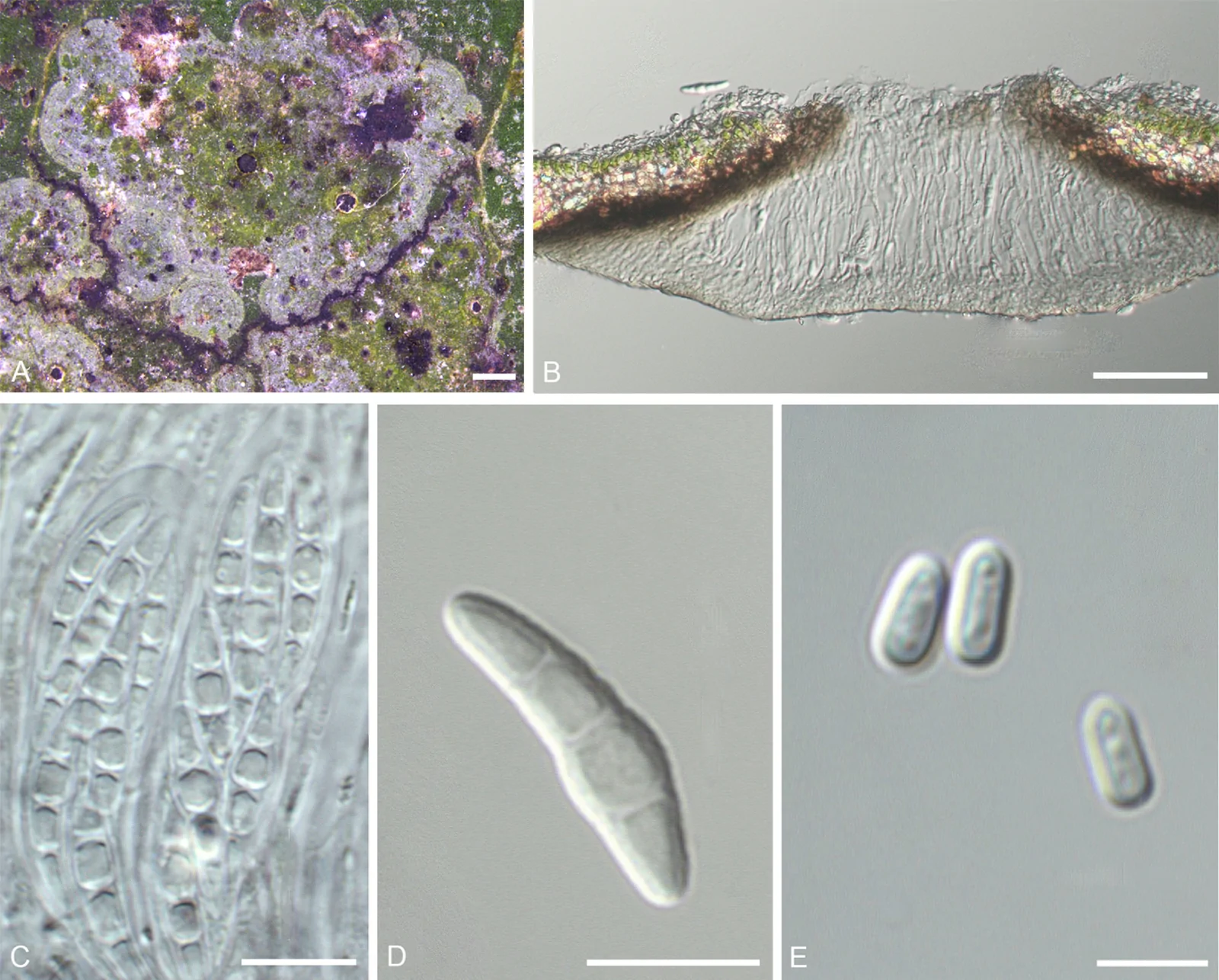
*Mazosia sinensis* sp. nov. **A** Thallus with ascomata (HMAS–L 152283). **B** Apothecium section (HMAS–L 152282). **C** Asci (HMAS–L 152282). **D** Ascospore (HMAS–L 152283). **E** Macroconidia (HMAS–L 152283). Scale bars: 0.4 mm (**A**); 50 μm (**B**); 10 μm (**C, D**); 5 μm (**E**).

##### Chemistry.

No substances detected by TLC.

##### Habitat and distribution.

On unidentified angiosperm leaves in tropics; known only from the type locality, Hainan.

##### Additional material examined.

CHINA • Hainan Province, Ledong County, Jianfengling, alt. ca. 953 m, on living leaves, 9 Dec. 2019, Z.T. Yao HN20192602-1 (HMAS–L 153579), HN20192615-2 (HMAS–L 153583), HN20192617-4 (HMAS–L 152217); alt. ca. 1049 m, on living leaves, 9 Dec. 2019, Z.T. Yao HN20192654-1 (HMAS–L 152234); alt. ca. 631 m, on living leaves, 10 Dec. 2019, Z.T. Yao HN20192661-1 (HMAS–L 152235); alt. ca. 692 m, on living leaves, 10 Dec. 2019, Z.T. Yao HN20192711-2 (HMAS–L 153594). Wuzhishan City, Wuzhi Mountain, alt. ca. 743 m, on living leaves, 12 Dec. 2019, Z.T. Yao HN20192759-2 (HMAS–L 156347), HN20192760-2 (HMAS–L 152273); alt. ca. 784 m, on living leaves, 12 Dec. 2019, Z.T. Yao HN20192778-1 (HMAS–L 153616), HN20192782-1 (HMAS–L 152282).

##### Notes.

Among other *Mazosia* species with brown verrucae, *M. bambusae* is most similar to the new species, but it has a larger thallus (5–20 µm) and larger apothecia (0.3–0.6 mm; Lücking 2008). *Mazosia tenuissima* is also similar to this new species, but its thallus often with indistinct prothallus line and the verrucae are always of thallus color (Lücking 2008). *Mazosia aptrootii* can be distinguished by its pilose thallus and 3–5-septate ascospores (Sipman 1991). *Mazosia lueckingii* and *M. quadriseptata* both have larger ascospores than the new species (Singh and Pinokiyo 2008). Another similar species is *M. pseudoaptrootii*, but it differs by its pale brownish yellow, larger thallus (2–11 mm) with distinct hairs, and by its longer asci (55–70 × 10–15 µm; see above).

#### Mazosia straminea

Taxon classificationAnimaliaArthonialesRoccellaceae

S.H. Jiang, Z.T. Yao, J.C. Wei & Lücking sp. nov.

0A216A4D-9E5C-54A7-8493-D610475B8A5F

852582

Fig. 16

##### Etymology.

The epithet refers to the grayish yellow brown thallus.

##### Typus.

CHINA • Hainan Province, Lingshui County, Diaoluo Mountain National Nature Reserve, alt. ca. 928 m, on living leaves, 14 Dec. 2019, Z.T. Yao HN20192871-2 (holotype HMAS–L 152300).

##### Diagnosis.

The new species is similar to *Mazosia phyllosema* in the size of ascospores, but can be distinguished by its greyish yellow brown thallus, higher ascomata (125–140 µm vs. 60–80 µm), and psoromic acid detected by TLC.

##### Description.

Thallus crustose, foliicolous, grayish green to yellow brown, rough, 5–11 mm across, 18–23 µm thick, sometimes with a brown prothallus, without verrucae. Photobiont *Phycopeltis* sp., cells rectangular, 7.5–20 × 7.5–10 µm. Ascomata rounded, 0.3–0.5 mm diam., and 125–140 µm high; disc black, often with narrow fissures around the edge of the disc, white powder under the thallus; when immature, the disc often flattened, covered by the thallus and not visible. Excipulum dark brown, 12.5–15 µm thick. Hypothecium colorless, 12.5–20 µm high. Hymenium colorless, 100–115 µm high. Asci clavate, 55–65 × 12.5–15 µm, 8-spored. Ascospores fusiform, colorless, 3-septate, 20–25 × 4.5–5.5 µm, the second cell is slightly enlarged. Pycnidia low conical, most are covered by thallus, with small black spots on the top. Macroconidia bacillar, non-septate, 4.5–5 × 2–2.5 µm. Microconidia not seen.

**Figure 16.**
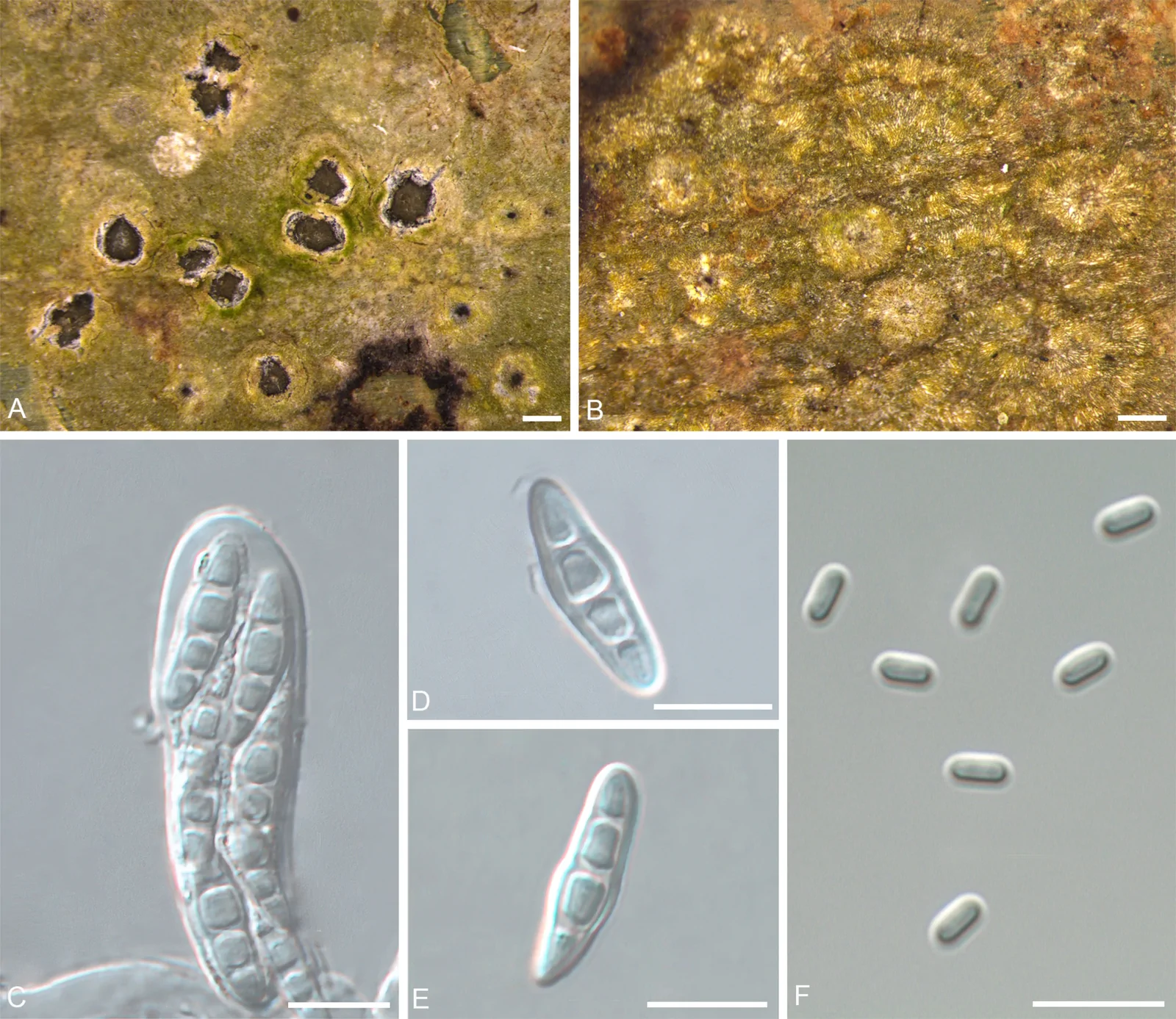
*Mazosia straminea* sp. nov. (HMAS–L 152300). **A, B** Thallus with ascomata. **C** Asci. **D, E** Ascospores. **F** Macroconidia. Scale bars: 0.2 mm (**A, B**); 10 μm (**C–F**).

##### Chemistry.

Psoromic acid.

##### Habitat and distribution.

On unidentified angiosperm leaves in tropics; known only from the type locality, Hainan.

##### Additional material examined.

CHINA • Hainan Province, Lingshui County, Diaoluo Mountain National Nature Reserve, alt. ca. 928 m, on living leaves, 14 Dec. 2019, Z.T. Yao HN20192872-1 (HMAS–L 152301).

##### Notes.

The new species is distinguishable from others by the greyish yellow-brown thallus, the special ontogeny of the ascomata, the 3-septate ascospores, and the psoromic acid chemistry. It is similar to *Mazosia phyllosema* in the size of ascospores, but the latter differs in the pale green thallus, lower ascomata (60–80 µm high), and no substances detected by TLC (Lücking 2008). *Mazosia paupercula* also has a smooth thallus, but its ascospores are 3–7-septate and larger (25–35 × 3.5–5.5 µm; Lücking 2008).

#### Mazosia verrucosa

Taxon classificationAnimaliaArthonialesRoccellaceae

S.H. Jiang, Z.T. Yao, J.C. Wei & Lücking sp. nov.

301CF310-8578-5047-9CFD-276B3C91A27D

852583

Fig. 17

##### Etymology.

The epithet refers to the verrucose thallus.

##### Typus.

CHINA • Hainan Province, Changjiang County, Bawangling National Nature Reserve, alt. ca. 697 m, on living leaves, 7 Dec. 2019, Z.T. Yao HN20192509-1 (holotype HMAS–L 152204).

##### Diagnosis.

The new species is similar to *Mazosia pseudomelanophthalma*, but the thallus (0.3–2 mm vs. 1.5–7 mm) and verrucae (0.03–0.05 mm vs. 0.05–0.1 mm diam.) are smaller, and the asci are narrower (9.5–11.5 µm vs. 12–15 µm).

##### Description.

Thallus crustose, foliicolous, yellow green, continuous, rounded, without prothallus, often singly clustered together to form larger patches with dark brown borders between adjacent thallus, 0.3–2 mm across, 7.5–12.5 µm thick, verrucae, glabrous; verrucae 0.03–0.05 mm, with the same color of the thallus. Photobiont *Phycopeltis* sp., cells rectangular, 8–12 × 3–5 µm, in radiate plates. Ascomata rounded, 0.05–0.17 mm diam., and 75–100 µm high; disc black, translucent when moistened and gently sloping outwards. Excipulum dark brown, 10–15 µm thick. Hypothecium pale brown, 12.5–22.5 µm high. Hymenium colorless, 65–75 µm high. Asci clavate, 40–53 × 9.5–11.5 µm, 8-spored. Ascospores fusiform, colorless, 3-septate, 13.5–23 × 3.5–4.5 µm, the second cell is slightly enlarged. Pycnidia low conical, 0.02–0.03 mm diam. Macroconidia bacillar, non-septate, 5–6.5 × 1.5–2 µm. Microconidia colorless, non-septate, 2.5–3 × 0.8–1 µm.

**Figure 17.**
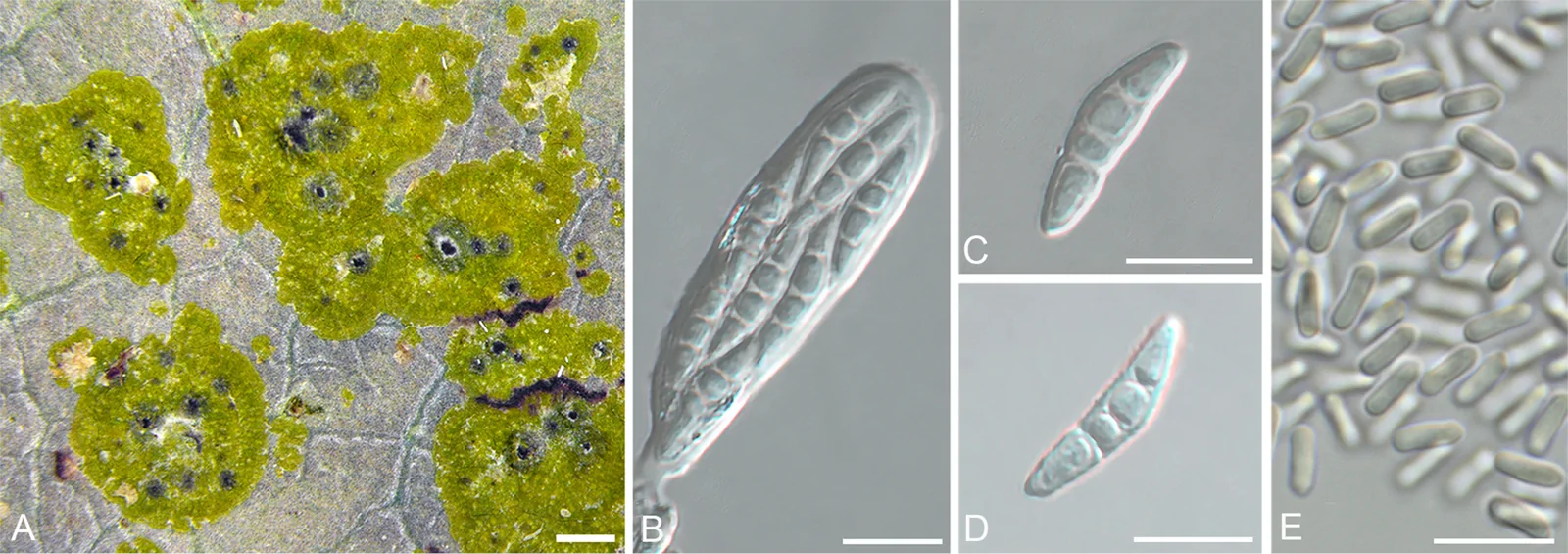
*Mazosia verrucosa* sp. nov. (HMAS–L 152204). **A** Thallus with ascomata. **B** Asci. **C, D** Ascospores. **E** Macroconidia. Scale bars: 0.4 mm (**A**); 10 μm (**B–E**).

##### Chemistry.

No substances detected.

##### Habitat and distribution.

On unidentified angiosperm leaves in tropics; known only from the type locality, Hainan.

##### Additional material examined.

CHINA • Hainan Province, Changjiang County, Bawangling National Nature Reserve, alt. ca. 700 m, on living leaves, 4 Sep. 2017, S.H. Jiang HN20171678-2 (HMAS–L 152096), HN20171715-1 (HMAS–L 152101), HN20171791-1 (HMAS–L 152177); alt. ca. 762 m, on living leaves, 7 Dec. 2019, Z.T. Yao HN20192508 (HMAS–L 152203); alt. ca. 697 m, on living leaves, 7 Dec. 2019, Z.T. Yao HN20192510-1 (HMAS–L 152205), HN20192511-1 (HMAS–L 152206), HN20192513-1 (HMAS–L 152207); alt. ca. 632 m, on living leaves, 7 Dec. 2019, Z.T. Yao HN20192529 (HMAS–L 152208), HN20192539-1 (HMAS–L 152209); alt. ca. 437 m, on living leaves, 8 Dec. 2019, Z.T. Yao HN20192540-1 (HMAS–L 147806), HN20192542-1 (HMAS–L 152210); alt. ca. 894 m, on living leaves, 8 Dec. 2019, Z.T. Yao HN20192579-1 (HMAS–L 152211), HN20192583 (HMAS–L 152212).

##### Notes.

*Mazosia verrucosa* is most similar to *M. pseudomelanophthalma*, but the latter differs by the larger thallus (1.5–7 mm), the larger verrucae (0.05–0.1 mm diam.), and the broader asci (12–15 µm; see above). This new species is also similar to *Mazosia melanophthalma*, but the latter has the larger verrucae (0.05–0.1 mm), larger ascomata (0.3–0.7 mm diam.), and wider macroconidia (5–8 × 2–3 µm; Lücking 2008). Another related species is *M. pseudobambusae*, but it often has a larger, pale brown thallus with dark brown prothallus, larger ascospores (20–28 × 4–5 µm), and wider macroconidida (4–6 × 2–2.5 µm; Lücking 2008). *Mazosia gelatinospora* differs by its characterized ascospores having a gelatinous sheath (see above).

### World key to the known species of *Mazosia*

#### Skeleton key

Corticolous, thallus verrucose **4**

Corticolous, thallus smooth to uneven **8**

Lichenicolous on foliicolous lichens **11**

Foliicolous, sorediate **12**

Foliicolous, thallus with radiating ridges **13**

Foliicolous, thallus verrucose and finely pilose **16**

Foliicolous, thallus verrucose but otherwise glabrous; ascospores (3–)5–9-septate **25**

Foliicolous, thallus verrucose but otherwise glabrous; ascospores regularly 3-septate **31**

Foliicolous, thallus smooth and finely pilose **42**

Foliicolous, thallus smooth, glabrous **46**

#### Main key

**Table:** 

1	Corticolous; ascospores 3(–5)-septate	**2**
–	Foliicolous or lichenicolous on foliicolous lichens; ascospores (1–)3(–9)-septate	**11**
2	Thallus distinctly byssoid	***Mazosia byssoidea* Lebreton & Ertz**
–	Thallus not byssoid	**3**
3	Thallus distinctly verrucose(-rugose)	**4**
–	Thallus smooth to uneven (to finely cracked)	**8**
4	Ascospores 3–5-septate, 20–28 × 5–6.5 µm; TLC with unknown substance; China	***M. pseudocorticola* (sp. nov.)**
–	Ascospores regularly 3-septate, 15–20(–25) × 4–6 µm; TLC no substan­ces	**5**
5	Apothecia distinctly erumpent, with steeply sloping margins	**6**
–	Apothecia immersed, with flush margin	**7**
6	Apothecial margins verrucose; Neotropics	***M. viridescens* (Fée) Aptroot & M. Cáceres**
–	Apothecial margins smooth; Neotropics	***M. ocellata* (Nyl.) R.C. Harris**
7	Ascospores (15–)17–25 µm long; prothallus indistinct; Japan	***M. bruguierae* A. Sakata & H. Harada**
–	Ascospores 15–18 µm long; prothallus forming a blackish line; Neotropics	***M. carnea* (Eckfeldt) Aptroot & M. Cáceres**
8	Psoromic acid (P+ yellow); Tasmania	***M. corticola* Kantvilas**
–	TLC no substances (P–)	**9**
9	Ascospores 25–35 × 5–7 µm; apothecia immersed, with flush margins; Neotropics	***M. leptosticta* (Nyl.) Sparrius**
–	Ascospores 15–20 × 3–6 µm; apothecia erumpent, with sloping margins	**10**
10	Apothecia with steeply sloping margins; pycnidia sessile; ascospores 4–6 µm broad; Neotropics	***M. endonigra* M. Cáceres & Aptroot**
–	Apothecia with gently sloping margins; pycnidia unknown; ascospores 3–4 µm broad; Japan	***M. japonica* A. Sakata & H. Harada**
11	Lichenicolous on foliicolous *Mazosia* species	***M. adelphoparasitica* Matzer**
–	Autonomous lichens	**12**
12	Thallus with soralia only, developing in circular fashion from pycnidia; Neotropics	***M. sorediifera* Lücking & Matzer**
–	Thallus with apothecia, lacking soralia	**13**
13	Thallus with long, radiating ridges; ascospores 3-septate; psoromic acid (P+ yellow); pantropical	***M. rotula* (Mont.) A. Massal**.
–	Thallus smooth to verrucose; ascospores and chemistry variable	**14**
14	Thallus distinctly verrucose	**15**
–	Thallus smooth	**42**
15	Thallus in addition to verrucae with fine hairs formed by fungal hyphae	**16**
–	Thallus apart from verrucae glabrous	**24**
16	Thallus brownish, with dark brown verrucae and usually dark brown prothallus line	**17**
–	Thallus greenish, with thallus-colored to whitish verrucae and indistinct to rarely dark brown prothallus line	**19**
17	Ascospores (3–)5-septate; Australasia	***M. aptrootii* Sipman**
–	Ascospores regularly 3-septate	**18**
18	Ascospores 22–35 × 4–6.5 µm; prothallus line indistinct; TLC with unknown substance; China	***M. weii* Z.T. Yao, S.H. Jiang & Z.F. Jia**
–	Ascospores 18–25 × 4–5 µm; prothallus line dark; TLC no substance; China	***M. pseudoaptrootii* (sp. nov.)** (if thallus greenish rather than brownish and verrucae partly of thallus color, see *M. sinensis*)
19	Ascospores (19)–22–38 µm long, usually > 25 µm; verrucae often whitish or thallus otherwise pruinose	**20**
–	Ascospores 15–23(–26) µm long, usually < 25 µm; verrucae of thallus color or sometimes dark; thallus not pruinose	**23**
20	Ascospores irregularly 3–5-septate; thallus pruinose; China	***M. pruinata* (sp. nov.)**
–	Ascospores regularly 3-septate; thallus variable	**21**
21	Ascospores (19)25–38 µm long; thallus pruinose; China	***M. pseudopruinata* (sp. nov.)**
–	Ascospores 22–30(–33) µm long; thallus variable	**22**
22	Thallus pruinose; macroconidia 5–6 × 2.2–2.7 µm; China	***M. papillosa* (sp. nov.)**
–	Thallus non-pruinose; macroconidia 4–4.7 × 1.7–2.2 µm; China	***M. pseudotenuissima* (sp. nov.)**
23	Thallus with dark prothallus line; verrucae often partially dark; China	***M. sinensis* (sp. nov.)**
–	Thallus with indistinct prothallus line; verrucae always of thallus color; Neotropics	***M. tenuissima* Lücking & Matzer**
24	Ascospores (3–)5–9-septate	**25**
–	Ascospores regularly 3-septate	**31**
25	Ascospores 5–9-septate	**26**
–	Ascospores 3–5-septate	**29**
26	Ascospores 5(–7)-septate, 25–40 × 4–7 µm, 5–6 times as long as broad; thallus often pruinose; pantropical	***M. dispersa* (J. Hedrick) R. Sant**.
–	Ascospores 7–9-septate, 40–65 × 3–4.5 µm, 10–15 times as long as broad; thallus non-pruinose	**27**
27	Ascospores 9-septate; verrucae with a yellow pigment; Neotropics	***M. flavida* Aptroot**
–	Ascospores 7-septate; verrucae without pigment	**28**
28	Apothecia prominent, with thick, steeply sloping margins; ascospores 40–50 × 3–4 µm; psoromic acid (P+ yellow); Neotropics	***M. praemorsa* (Stirt.) R. Sant**.
–	Apothecia erumpent, with thin, gently sloping margins; ascospores 55–65 × 4–5 µm; TCL no substance (P–); Neotropics	***M. longispora* Lücking & Matzer**
29	Thallus brownish, with dark verrucae and dark prothallus line; ascospores irregularly 3–5-septate; India	***M. lueckingii* Kr.P. Singh & Pinokiyo**
–	Thallus greenish, with thallus-coloured verrucae and indistinct prothallus line; ascospores regularly 4-septate	**30**
30	Thallus verrucae with reddish pigment; ascospores 20–27 µm long; Neotropics	***M. rubropunctata* R. Sant**.
–	Thallus verrucae without pigment; ascospores 25–33 µm long; Australasia	***M. quadriseptata* Kalb & Vězda**
31	Thallus very coarsely verrucose; apothecia with thick, steeply sloping margin; psoromic acid (P+ yellow): Neotropics	***M. tumidula* (Müll. Arg.) Zahlbr**.
–	Thallus finely verrucose; apothecia with thin to rarely thickened, gently sloping to flush margin; TLC no or unknown substance (P–)	**32**
32	Thallus with a dark prothallus line	**33**
–	Thallus with indistinct prothallus line	**36**
33	Thallus brownish	**34**
–	Thallus greenish	**35**
34	Thallus verrucae dark	***M. bambusae* (Vain.) R. Sant**.
–	Thallus verrucae pale	***M. pseudobambusae* Kalb & Vězda**
35	Thallus verrucae somewhat radiating; macroconidia 6.5–7.5 × 2.2–2.5 µm; China	***M. dimorphoverrucosa* (sp. nov.)**
–	Thallus verrucae dispersed; macroconidia 4–5 × 1.5–2 µm; China	***M. intermedia* (sp. nov.)**
36	Ascospores 23–30 µm long; China	***M. pseudopapillosa* (sp. nov.)** (if thallus partly finely pilose, see *M. papillosa*)
–	Ascospores 14–23(–25) µm long	**37**
37	Thallus verrucae arranged in (somewhat) concentric circles	**38**
–	Thallus verrucae dispersed	**39**
38	Ascospores with gelatinous sheath; TLC no substances; China	***M. gelatinospora* (sp. nov.)**
–	Ascospores lacking gelatinous sheath; unknown substance; China	***M. centrica* (sp. nov.)**
39	Apothecia immersed, with thickened, flush margin; unknown substance; China	***M. hainanensis* Z.T. Yao, S.H. Jiang & Z.F. Jia**
–	Apothecia erumpent, with thin, gently sloping margin	**40**
40	Macroconidia 2–3 µm broad; pantropical	***M. melanophthalma* (Müll. Arg.) R. Sant**.
–	Macroconidia 1.5–2 µm broad	**41**
41	Asci 12–15 µm broad; China	***M. pseudomelanophthalma* (sp. nov.)**
–	Asci 9–12 µm broad; China	***M. verrucosa* (sp. nov.)**
42	Thallus with fine hairs formed by fungal hyphae	**43**
–	Thallus glabrous	**46**
43	Ascospores (3–)5–7-septate	**44**
–	Ascospores regularly 1- or 3-septate	**45**
44	Ascospores irregularly (3–)5–7-septate; unknown substance; China	***M. pseudopilosa* (sp. nov.)**
–	Ascospores regularly 5-septate; TLC no substance; Australia	***M. tomentifera* Vězda & Lumbsch**
45	Ascospores 1-septate, 10–14 µm long; Neotropics	***M. uniseptata* Lücking**
–	Ascospores 3-septate, 17–27 µm long; Neotropics	***M. pilosa* Kalb & Vězda**
46	Ascospores irregularly (3–)5(–7)-septate; pantropical	***M. paupercula* (Müll. Arg.) R. Sant**.
–	Ascospores regularly 3-septate	**47**
47	Ascospores 10–14 µm long; pycnidia conical; Neotropics	***M. conica* Sérus**.
–	Ascospores 15–25 µm long; pycnidia wart-shaped	**48**
48	Psoromic acid (P+ yellow); China	***M. straminea* (sp. nov.)**
–	TLC no substance (P–); pantropical	***M. phyllosema* (Nyl.) Zahlbr**.

## Supplementary Material

XML Treatment for Mazosia centrica

XML Treatment for Mazosia dimorphoverrucosa

XML Treatment for Mazosia gelatinospora

XML Treatment for Mazosia intermedia

XML Treatment for Mazosia papillosa

XML Treatment for Mazosia pruinata

XML Treatment for Mazosia pseudoaptrootii

XML Treatment for Mazosia pseudocorticola

XML Treatment for Mazosia pseudomelanophthalma

XML Treatment for Mazosia pseudopapillosa

XML Treatment for Mazosia pseudopilosa

XML Treatment for Mazosia pseudopruinata

XML Treatment for Mazosia pseudotenuissima

XML Treatment for Mazosia sinensis

XML Treatment for Mazosia straminea

XML Treatment for Mazosia verrucosa

## Additional information

### Artificial Intelligence (AI) use

The authors accept full responsibility for the content of the manuscript, including the disclosure of any use of AI.

No AI tools were used in the preparation of this manuscript.

### Adherence to national and international regulations

All the fungal strains used in this study have been legally obtained, respecting the Convention on Biological Diversity (Rio Convention).

### Author ORCIDs

S.-H. Jiang https://orcid.org/0000-0002-1806-7356

M.E.S. Cáceres https://orcid.org/0000-0002-5612-1309

A. Aptroot https://orcid.org/0000-0001-7949-2594

R. Lücking https://orcid.org/0000-0002-3431-4636

### Data availability

All data generated or analyzed during this study are included in this published article or in the supplementary materials. All sequences during this study have been submitted to GenBank.

## Supplementary materials

Supplementary material 1 Sequence alignments Data type fas Explanation note Sequence alignments of ITS, LSU and mtSSU loci of *Mazosia* used for Fig. 1. https://binary.pensoft.net/file/1744086This dataset is made available under the Open Database License (http://opendatacommons.org/licenses/odbl/1.0/). The Open Database License (ODbL) is a license agreement intended to allow users to freely share, modify, and use this Dataset while maintaining this same freedom for others, provided that the original source and author(s) are credited. Shu-Hua Jiang
